# Novel insights from meta-analysis: the efficacy of ginsenosides in non-alcoholic fatty liver disease

**DOI:** 10.3389/fphar.2025.1564852

**Published:** 2025-05-27

**Authors:** Liyuan Hao, Qing Peng, Shenghao Li, Xiaoyu Hu, Huimin Yan

**Affiliations:** ^1^ School of Clinical Medicine, Chengdu University of Traditional Chinese Medicine, Chengdu, Sichuan, China; ^2^ Department of Infectious Diseases, Hospital of Chengdu University of Traditional Chinese Medicine, Chengdu, Sichuan, China; ^3^ Department of Integrated Traditional Chinese and Western Medicine Oncology, The Fourth Hospital of Hebei Medical University, Shijiazhuang, Hebei, China; ^4^ Clinical Research Center, Shijiazhuang Fifth Hospital, Shijiazhuang, Hebei, China

**Keywords:** ginsenoside, animal model, NAFLD, preclinical studies, meta-analysis

## Abstract

**Background:**

The global prevalence of non-alcoholic fatty liver disease (NAFLD) has surged, largely driven by modern lifestyle changes and dietary shifts. As these factors profoundly impact human health, exploring effective therapeutic strategies for NAFLD has become a pressing medical concern. Previous studies have suggested that ginsenosides may offer a potential treatment approach for NAFLD by reducing oxidative stress and controlling inflammation. However, its efficacy and safety remain unclear. Therefore, the aim of this systematic review and meta-analysis is to evaluate the role of ginsenosides in the treatment of NAFLD.

**Methods:**

We searched for relevant studies published through September 2024, including databases such as PubMed, Embase, Web of Science, China National Knowledge Infrastructure (CNKI), and Wanfang Data. The SYstematic Review Center for Laboratory animal Experimentation (SYRCLE) Animal Experiment Bias Risk Assessment Tool was used to evaluate the quality of the literature. Subsequently, Review Manager (RevMan, version 5.3) and STATA 15 software was utilized for data analysis.

**Results:**

Finally, 30 studies involving a total of 604 animals were included in the analysis. The results showed that, compared with the model group, ginsenosides significantly reduces total cholesterol (TC), triglycerides (TG), alanine aminotransferase (ALT), aspartate aminotransferase (AST), low-density lipoprotein cholesterol (LDL), body weight, liver weight, liver index, serum insulin, tumor necrosis factor-α (TNF-α), interleukin-1 (IL-1), interleukin-6 (IL-6) and NAFLD Activity Score (NAS). Due to the high heterogeneity, we conducted subgroup analyses of the main results ALT, AST, TC and TG by animal strains, modeling methods, administration methods, ginsenoside dosages and types of ginsenosides. The results showed that the heterogeneity of TC may be derived from differences in modeling methods. The results showed that the heterogeneity of TC may be derived from differences in modeling methods.

**Conclusion:**

In this study, we summarized the molecular mechanism of ginsenosides in regulating NAFLD, mainly focusing on inhibiting inflammation and oxidative stress, improving insulin sensitivity, and regulating intestinal flora. Preclinical evidence indicates that ginsenosides represent a novel therapeutic avenue for NAFLD. The mechanism of ginsenosides in treating NAFLD may involve anti-inflammation, antioxidation, improving insulin resistance, and regulating intestinal flora. However, the inclusion of studies with low methodological quality and the existence of publication bias may undermine the validity of the results. To fully elucidate the mechanisms underlying the therapeutic effects of ginsenosides, future research should employ more rigorous experimental designs and conduct comprehensive investigations.

**Systematic Review Registration:**

https://www.crd.york.ac.uk/PROSPERO/.

## Introduction

Non-alcoholic fatty liver disease (NAFLD) has emerged as a rapidly spreading global health issue, afflicting approximately one-fourth of the adult population worldwide (Guo et al., 2022). As the hepatic manifestation of metabolic syndrome, NAFLD encompasses a continuum of liver disorders, ranging from simple steatosis to more severe conditions such as non-alcoholic steatohepatitis (NASH), fibrosis, cirrhosis, and even hepatocellular carcinoma (HCC) (Huby and Gautier, 2022). Currently, there are no approved drugs for the treatment of NAFLD and NASH, and there are other drugs clinically available for the treatment of NAFLD and NASH-related problems, which increases the need to understand the pathogenesis and progression of NAFLD and NASH to develop new treatment strategies. Recently, research has increasingly focused on identifying novel therapies for NAFLD/NASH.

Ginseng is a rhizomatous plant and has long been used as a traditional herbal medicine for treating various physiological and pathological conditions (Lü et al., 2009). Ginseng contains different types of bioactive components such as ginsenosides, polysaccharides, etc. (Attele et al., 1999). Among these, ginsenosides (i.e., steroidal saponins) have been identified as the principal bioactive compounds responsible for modulating numerous biological and physiological processes in the human body. Accumulating evidence indicates that ginsenosides exert potent pharmacological effects in various diseases by suppressing oxidative stress, inflammation, and fibrosis (Zhao et al., 2023; Yi, 2021). An increasing number of studies have shown that ginseng and ginsenosides play pharmacological roles in the pathogenesis and progression of NAFLD and NASH. Ginsenoside Rg3 has been reported to reduce liver inflammation and fibrosis by regulating the NF-κB signaling pathway, thereby preventing the progression of NAFLD to NASH (Lee et al., 2020). Ginsenoside Rg3 reduces insulin resistance and lipotoxicity associated with obesity through the STAT5-PPARγ pathway (Lee et al., 2017). In addition, ginsenoside Rg1 protects the liver from age-related fatty liver disease by maintaining hepatic orkhead box protein O1 (FOXO1) activity nd reducing inflammation (Qi et al., 2020). Given the promising efficacy of ginsenosides, researchers have increasingly focused on exploring their applications in NAFLD treatment. Although there is sufficient evidence detailing the effects of ginsenosides on many signaling pathways and cellular functions in NAFLD, an overall view of their mechanism of action remains elusive. Therefore, this systematic review aims to synthesize existing research reports, summarizing the key findings on the therapeutic effects of ginsenosides in NAFLD, as well as their pharmacological actions and potential underlying mechanisms.

## Materials and methods

### Literature search and review strategy

This report is registered in PROSPERO (NO. CRD42024611305). Five databases, PubMed, Web of Science, Embase, China National Knowledge Infrastructure (CNKI) and Wanfang Data were searched from the establishment of the database to September 2024. Searches were conducted using MeSH terms and free-text words appropriately adapted for each database. The medical subject terms (MeSH) and free terms used for database searches are “Non-alcoholic Fatty Liver Disease” or “Non-alcoholic Fatty Liver Disease” or “Fatty Liver, Nonalcoholic” or “Fatty Livers, Nonalcoholic” or “Liver, Nonalcoholic Fatty” or “Livers, Nonalcoholic Fatty” or “Nonalcoholic Fatty Liver” or “Nonalcoholic Fatty Livers” or “NAFLD” or “Nonalcoholic Fatty Liver Disease” or “Nonalcoholic Steatohepatitis” or “Nonalcoholic Steatohepatitides” or “Steatohepatitides, Nonalcoholic” or “Steatohepatitis, Nonalcoholic” and “Ginsenosides” or “Ginsenoside” or “Panaxosides” or “Sanchinosides”.

### Inclusion and exclusion criteria

The following inclusion criteria should be met: 1) the experiment was based on the NAFLD/NASH model only; 2) only the ginsenoside group was included or it received ginsenoside; 3) the included studies consist of a model group and ginsenoside group; 4) the primary endpoints were as follows: total cholesterol (TC), triglycerides (TG), alanine aminotransferase (ALT), aspartate aminotransferase (AST), high-density lipoprotein cholesterol (HDL), low-density lipoprotein cholesterol (LDL), NAFLD Activity Score (NAS), and the secondary endpoints were as follows: animal body weight, liver weight, liver index, serum insulin, tumor necrosis factor-α (TNF-α), interleukin-1 (IL-1), interleukin-6 (IL-6).

The exclusion criteria of this study were as follows: 1) Animal experiments are not included; 2) Review and/or meta-analysis; 3) Randomized controlled trials or clinical studies; 4) Ginseng extracts or mixtures, experimental group without ginsenosides and NAFLD/NASH or other ginsenosides and NAFLD/NASH measures; 5) Insufficient primary and secondary outcome data; 6) comment, conference, editorial, letter, reply; 7) Irrelevant or duplicate publication.

### Data extraction

All the searched articles were imported into EndNote X9, and duplicate articles were removed. Two researchers independently conducted literature collection according to the inclusion and exclusion criteria. Initially, titles and abstracts were selected to exclude irrelevant articles. After the preliminary screening, potentially eligible articles underwent full-text screening for final determination.

Two reviewers extracted the following information from the included studies: 1) Basic data: last name, first name of the initial author, and publication year; 2) Characteristics of experimental animals, including animal type, gender, sample size, age, and weight; 3) Modeling method and criteria for successful modeling; 4) Treatment information: administration method, source, duration, and dose of the intervention drug; 5) Outcome measures: analysis of indicators such as TC, TG, ALT, AST, HDL, LDL, animal body weight, liver weight, liver index, serum insulin, TNF-α, IL-1, IL-6 and NAFLD Activity Score (NAS). All outcome measures are continuous data; therefore, the mean and standard deviation are plotted for each intervention group. If the results are presented only in chart form, we try to contact the authors for more details. In the same experiment, if there were multiple doses in treatment group, the highest dose group was chosen. If the results were acquired at multiple time points, only the data of the last time point is collected for analysis. If there is no response, the GetData Graph Digitizer software is used for graphic quantification.

### Quality evaluation

The SYstematic Review Center for Laboratory animal Experimentation (SYRCLE) risk of bias assessment tool was used to assess methodological quality. The assessment includes ten items (Guo et al., 2022): sequence generation (Huby and Gautier, 2022). baseline characteristics (Lü et al., 2009). allocation concealment (Attele et al., 1999). random housing (Zhao et al., 2023). blinding of experimenters (Yi, 2021). random outcome assessment (Lee et al., 2020). blinding of outcome assessors (Lee et al., 2017). incomplete outcome data (Qi et al., 2020). selective outcome reporting (Cui et al., 2023). other sources of bias. In addition, the assessment score for each part is yes (low risk of bias), no (high risk of bias), and unclear (the risk of bias is insufficient to be evaluated from the reported details). In case of disagreement, it is resolved through consultation with the corresponding author.

### Statistical analysis

Data analysis was performed using Review Manager (RevMan, version 5.3) and STATA 15 software. Since the variable type data in this report is continuous, standardized mean differences (SMD) and 95% confidence intervals (CI) are used to represent the effect size. For certain outcomes that are fairly standard (AST, ALT, TC, TG, HDL and LDL), using weighted mean differences (MD) could make the results more clinically interpretable. Heterogeneity between studies was evaluated using I-squared (I^2^). A fixed-effects model is used to combine effect sizes for I^2^ ≤ 50%. I^2^ > 50% is considered to represent great heterogeneity, and a random effects model is used to combine effect sizes. Due to the high heterogeneity, we conducted subgroup analyses of the main results ALT, AST, TC and TG by ginsenoside dosages (≤40 mg/kg and >40 mg/kg, according to the median dose used in the included studies), modeling methods (HFD, HSFD and HSFD and CCl4), types of ginsenosides (protopanaxatriol (PPT), protopanaxadiol (PPD) and other), animal strains (mice and rats), administration methods (intraperitoneal injection and gavage). Sensitivity analysis is performed when there is a significant deviation in individual results. If there are at least 10 studies for each outcome, funnel plots and Egger’s test are used to assess potential publication bias. The Trim-and-fill method is used when there is publication bias. This study did not conduct a sensitivity analysis based on the quality of the studies, mainly because the included studies had excessively high heterogeneity in terms of design, samples, and measurement methods, making it difficult to adopt a unified standard for quality evaluation.

## Results

### Study inclusion

**FIGURE 1:**
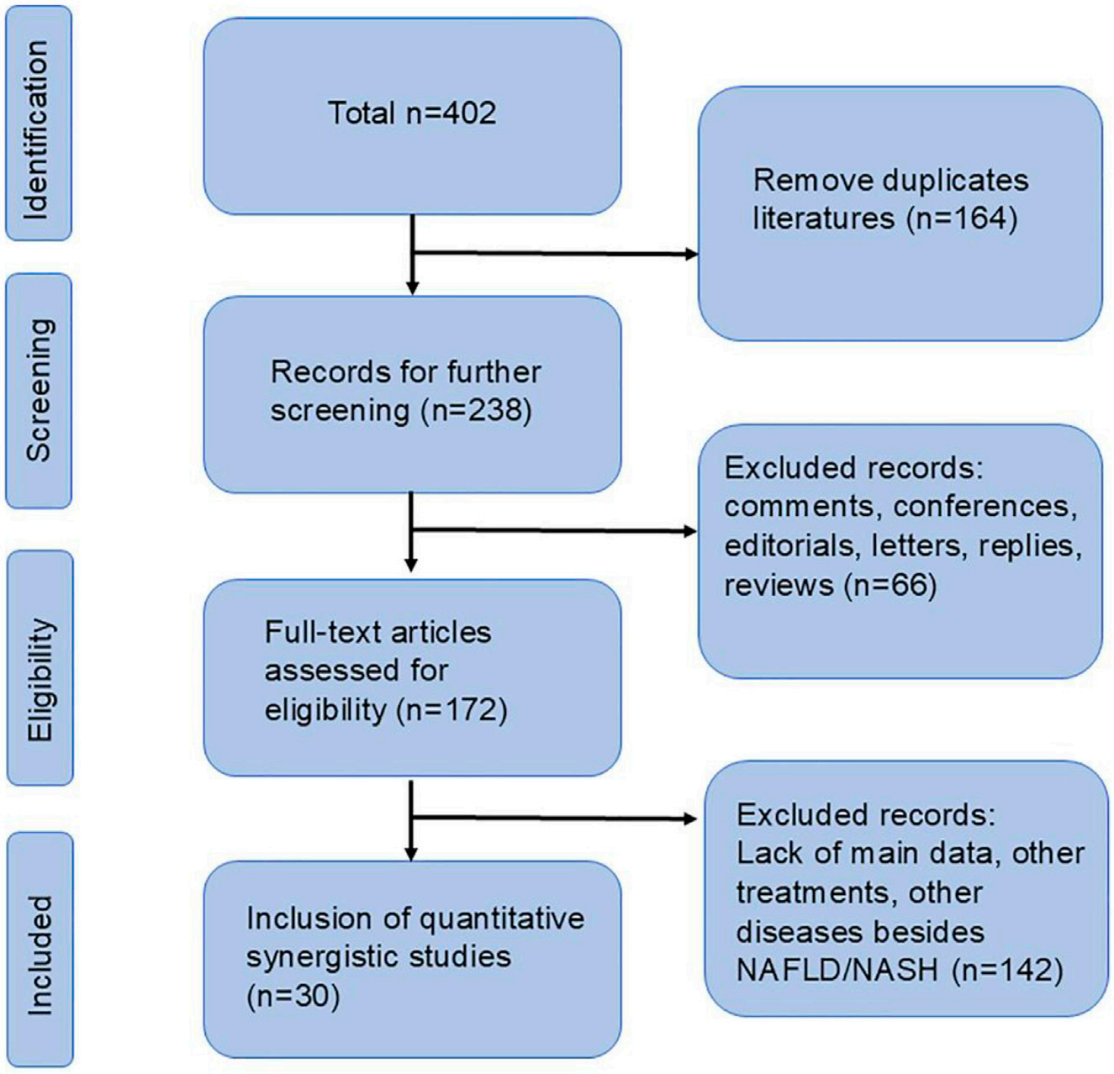
Flowchart for inclusion of studies.

According to our search strategy, a total of 402 relevant studies were identified. Among them, there were 61 in PubMed, 120 in Embase, 65 in Web of Science, 84 from CNKI and 72 from Wanfang. After removing duplicate documents, 164 documents were retained. By reading the titles, abstracts, and full texts, finally, according to our exclusion criteria, 30 studies were included for further analysis (Qi et al., 2020; Cui et al., 2023; Gu et al., 2021; Guo et al., 2023; Hou et al., 2020; Huang et al., 2017; Li and Fan, 2024; Li et al., 2024; Liang et al., 2021; Shi et al., 2024; Song et al., 2020; Xu YS. et al., 2018; Yang SM. et al., 2023; Yao, 2020; Zhang et al., 2024; Zhang et al., 2022; Cui, 2024; Gao et al., 2023; Han et al., 2019; He et al., 2021; Hong et al., 2018; Jiang, 2021; Liang et al., 2023; Liao and Xu, 2016; Peng et al., 2015; Ruan et al., 2020; Wang et al., 2021; Xiao Y. et al., 2019; Xie et al., 2023; Xu, 2018). The flow chart is shown in Figure 1.

#### Study characteristics

**FIGURE 2:**
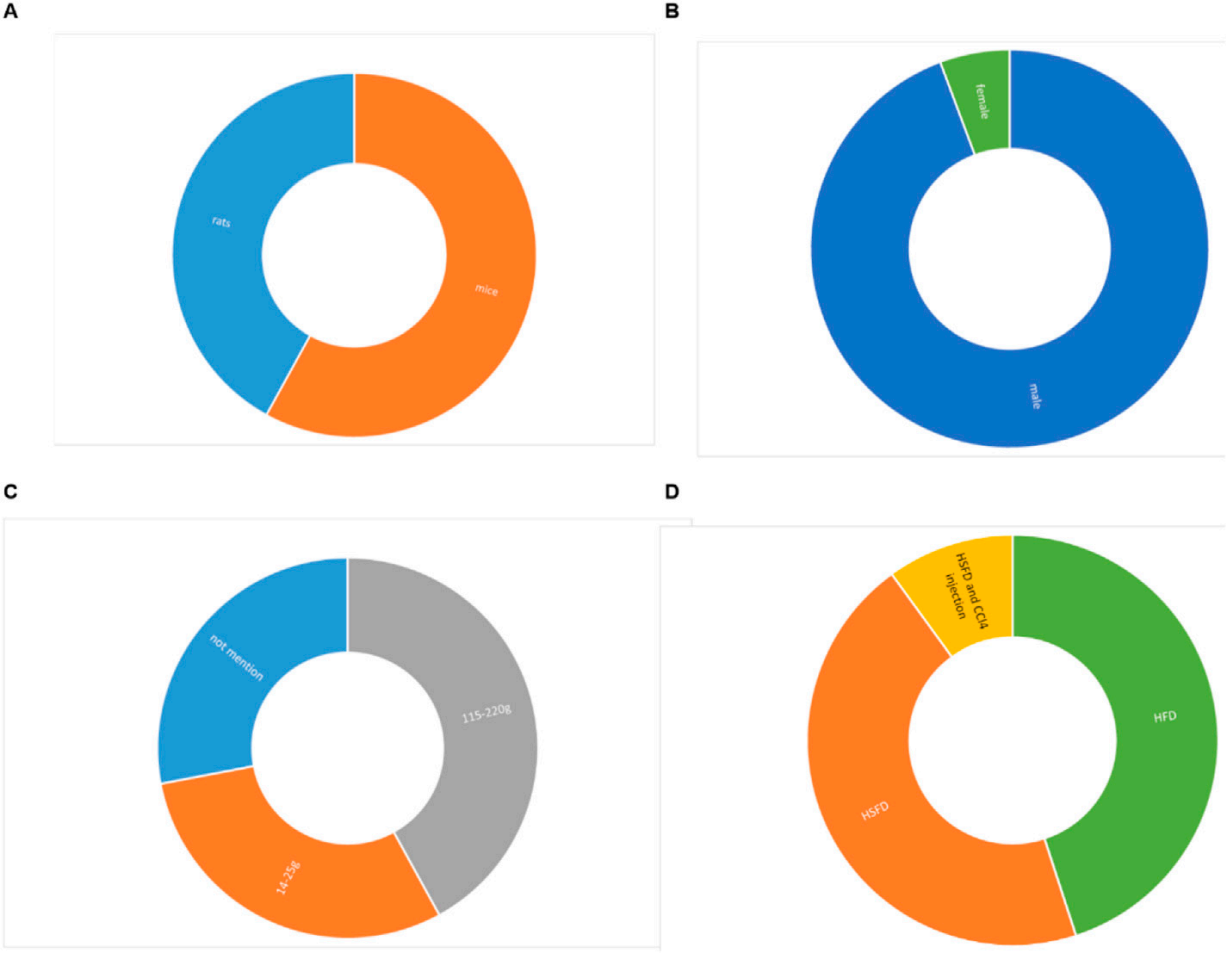
Study characteristics of studies. **(A)**. Animal strains, **(B)**. Animal sex, **(C)**. Weight, **(D)**. Modeling method.

A total of 30 studies were included in this research, which suggests that the protective effect of ginsenosides on NAFLD has attracted extensive attention in recent years. A total of 604 animals were included in these studies, with 304 in the model group and 300 in the ginsenoside group. The animal species utilized were rats and mice. Among them, 10 studies used rats (Gu et al., 2021; Hou et al., 2020; Gao et al., 2023; Jiang, 2021; Liang et al., 2023; Liao and Xu, 2016; Peng et al., 2015; Ruan et al., 2020; Xiao Y. et al., 2019; Xie et al., 2023), while 20 studies used all mice. In all studies, the weight of rats was 115–220g, and the weight of mice was 14–25 g. However, 10 studies did not report the animal weight or merely indicated the animals’ age in weeks. In terms of gender, only two studies (Xu YS. et al., 2018; Xu, 2018) utilized female mice, while the remaining 28 studies opted for male mice. The experimental group was given ginsenosides, and the model group was given normal saline or others. In terms of the modeling methods, three study (Guo et al., 2023; Li et al., 2024; Yang SM. et al., 2023) used HSFD combined with carbon tetrachloride (CCl4) for modeling, 16 studies used high-fat diet (HFD) for modeling, and the remaining 11 studies used high-sucrose and high-fat diet (HSFD) for modeling. The ginsenoside dosages in these studies varied across multiple levels, ranging from 1 to 200 mg/kg/day. The maximum dose was 200 mg/kg and lasted for a total of 12 weeks. The detailed information of the included studies is listed in Supplementary Table 1 and Figure 2.

#### Study quality

As shown in Table 1 and Supplementary Figure 1, we conducted a strict assessment of the quality of the included studies. The quality scores of all the studies fell within the range of four–five points. Among the 30 included studies, 28 studies reported the use of random grouping methods. The basic characteristics of animals between study groups were balanced in all studies. However, with respect to allocation concealment, seven studies failed to report any details regarding this aspect, and 21 studies were unclear. Concerning the blinding of experimenters, it remained ambiguous in 17 studies as to whether the experimenters were blinded during the conduct of the experiments. 27 studies did not report random for outcome assessment, and 22 studies did not report blinding of outcome assessors. In addition, no incomplete experimental data or selective reporting was found. These studies had no biases from other sources.

**TABLE 1:** Risk of bias of included studies.

Id	Sequence generation	Baseline characteristics	Allocation concealment	Random housing	Blinding of experimentalists	Random for outcome assessment	Blinding of outcome assessors	Incomplete outcome data	Selective outcome reporting	Other biases
Cui et al. (2023) (10)	-	+	-	+	-	-	-	+	+	+
Cui. (2024) (25)	?	+	?	+	?	-	?	+	+	+
Gao et al. (2023) (26)	?	+	?	+	?	?	?	+	+	+
Gu et al. (2021) (11)	?	+	?	+	?	-	-	+	+	+
Guo et al. (2023) (12)	?	+	?	+	?	-	-	+	+	+
Han et al. (2019) (27)	+	+	+	+	?	-	-	+	+	+
He et al. (2021) (28)	?	+	?	+	?	-	-	+	+	+
Hong et al. (2018) (29)	?	+	?	+	?	-	-	+	+	+
Hou et al. (2020) (13)	?	+	?	+	?	?	-	+	+	+
Huang et al. (2017) (14)	-	+	-	+	-	-	-	+	+	+
Jiang. (2021) (30)	?	+	?	+	-	-	-	+	+	+
Li et al. (2024) (15)	?	+	?	+	-	-	-	+	+	+
Li et al. (2024) (16)	-	+	-	+	-	-	?	+	+	+
Liang et al. (2021) (17)	?	+	?	+	-	-	?	+	+	+
Liang et al. (2023) (31)	?	+	?	+	?	-	-	+	+	+
Liao and Xu. (2016) (32)	-	+	-	+	-	-	-	+	+	+
Peng et al. (2015) (33)	?	+	?	+	?	-	-	+	+	+
Qi et al. (2020) (9)	?	+	?	+	-	?	-	+	+	+
Ruan et al. (2020) (34)	?	+	?	+	?	-	-	+	+	+
Shi et al. (2024) (18)	?	+	?	+	-	-	-	+	+	+
Song et al. (2020) (19)	?	+	?	-	-	-	-	+	+	+
Wang et al. (2021) (35)	?	+	?	+	?	-	-	+	+	+
Xiao et al. (2019a) (36)	?	+	?	+	?	-	-	+	+	+
Xie et al. (2023) (37)	?	+	?	+	-	-	-	+	+	+
Xu et al. (2018a) (20)	?	+	?	+	-	-	?	+	+	+
Xu et al. (2018a) (38)	+	+	+	+	-	-	-	+	+	+
Yang et al. (2023a) (21)	?	+	?	+	-	-	?	+	+	+
Yao. (2020) (22)	-	+	-	+	-	-	-	+	+	+
Zhang et al. (2022) (24)	-	+	-	+	-	-	?	+	+	+
Zhang et al. (2024) (23)	-	+	-	-	-	-	?	+	+	+

Note: +, low risk of bias; −, high risk of bias; ? unclear risk of bias.

### Effect of ginsenoside treatment on NAFLD

#### Primary outcome measure

##### Analysis of lipid content

**FIGURE 3:**
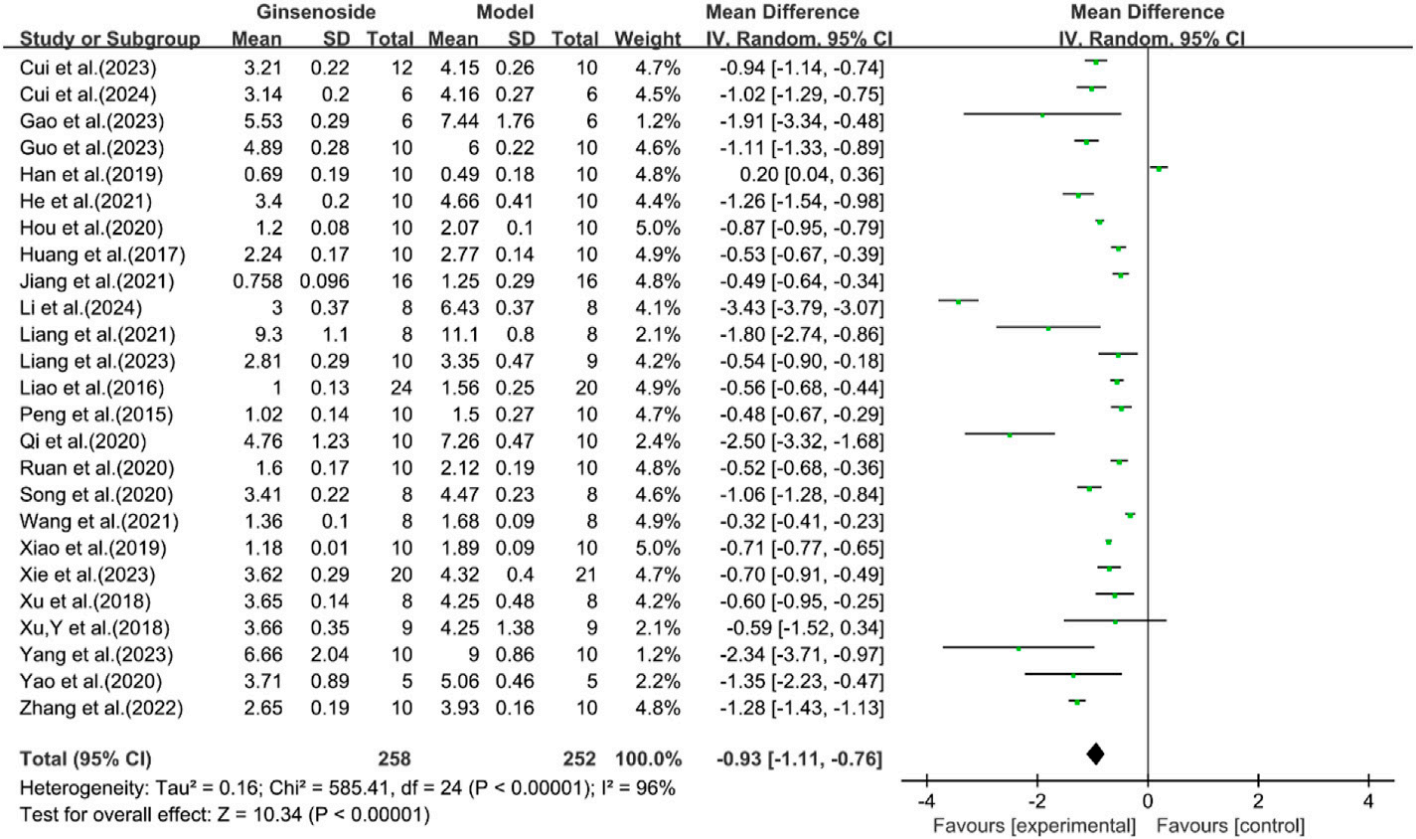
Forest plot: effect of ginsenosides on TC level.

A total of 25 studies reported the effect of ginsenosides on TC levels. The pooled results showed that compared with the model group, ginsenoside group significantly decreased TC levels [n = 510, MD = -0.93 [-1.11, −0.76], *P* < 0.05; heterogeneity: I^2^ = 96%, *P* < 0.00001] (Figure 3).

**FIGURE 4:**
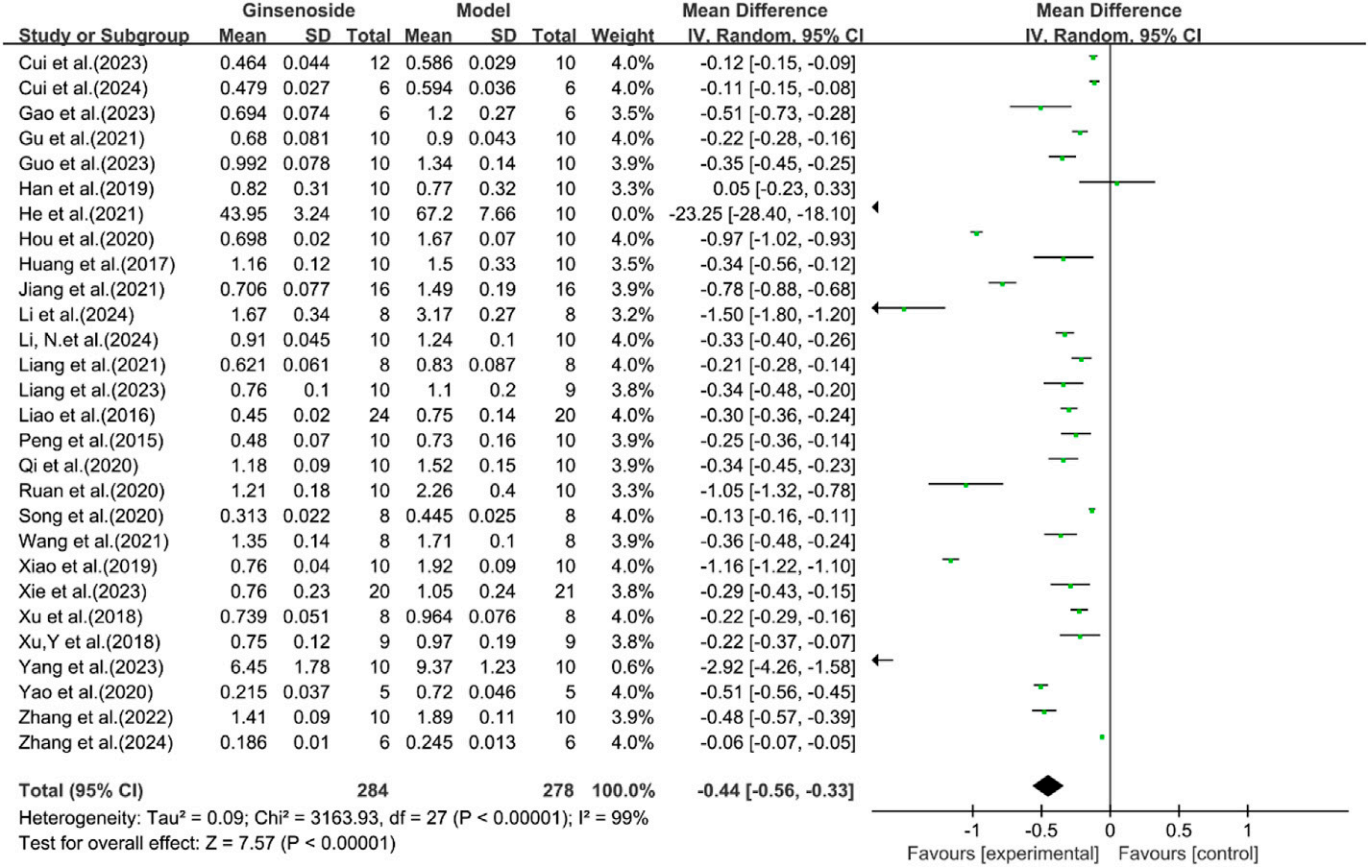
Forest plot: effect of ginsenosides on TG level.

A total of 28 studies reported the effect of ginsenosides on TG levels. The pooled results showed that compared with the model group, ginsenoside group significantly decreased TG levels [n = 562, MD = −0.44 [-0.56, −0.33], *P* < 0.05; heterogeneity: I^2^ = 99%, *P* < 0.00001] (Figure 4).

**FIGURE 5:**
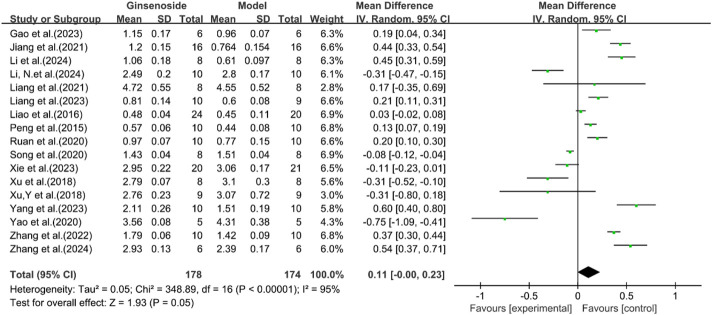
Forest plot: effect of ginsenosides on HDL level.

A total of 17 studies reported the effect of ginsenosides on HDL levels. The pooled results showed that there was no significant difference in HDL levels between the two groups [n = 352, MD = 0.11 [-0.00, 0.23], *P* = 0.05; heterogeneity: I^2^ = 95%, *P* < 0.00001] (Figure 5).

**FIGURE 6:**
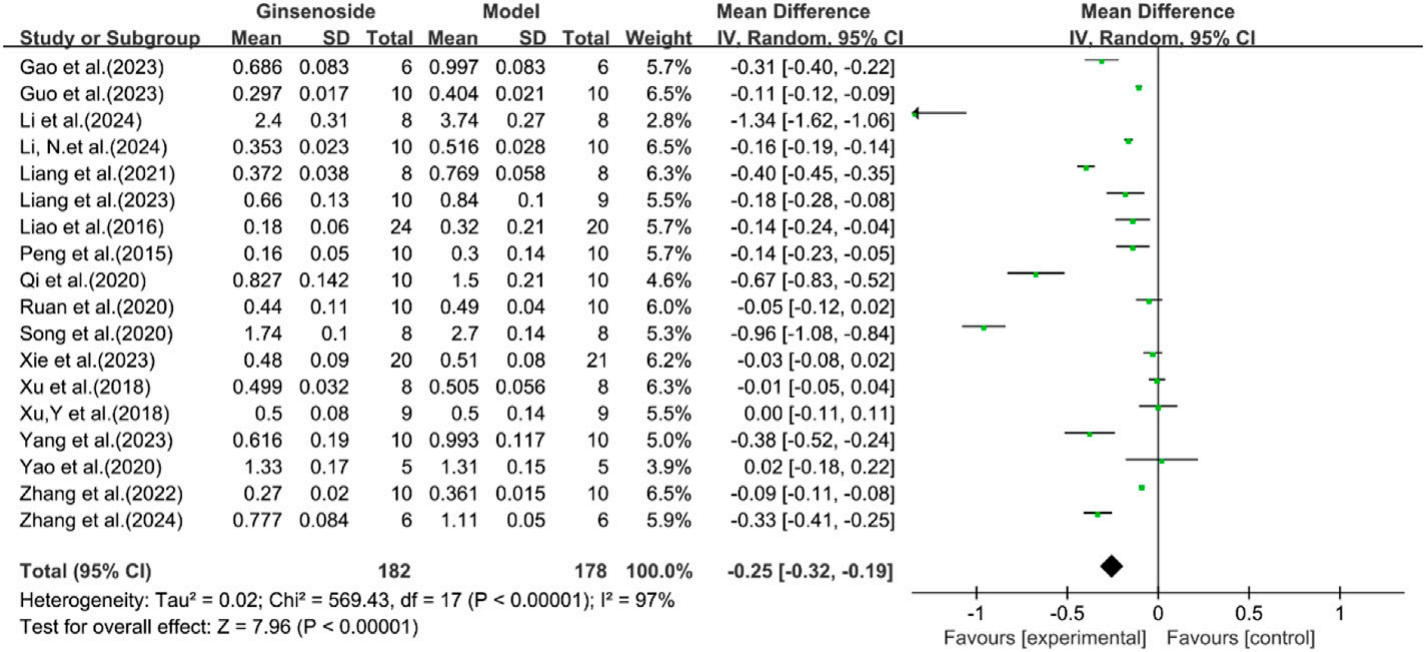
Forest plot: effect of ginsenosides on LDL level.

A total of 18 studies reported the effect of ginsenosides on LDL levels. The pooled results showed that two studies (Yao, 2020; Xu et al., 2018a) showed no significant difference in LDL levels between the two groups. The remaining studies indicated that compared with the model group, ginsenoside group significantly decreased LDL levels [n = 360, MD = −0.25 [-0.32, −0.19], *P* < 0.05; heterogeneity: I^2^ = 97%, *P* < 0.00001] (Figure 6).

#### Histological changes

**FIGURE 7:**
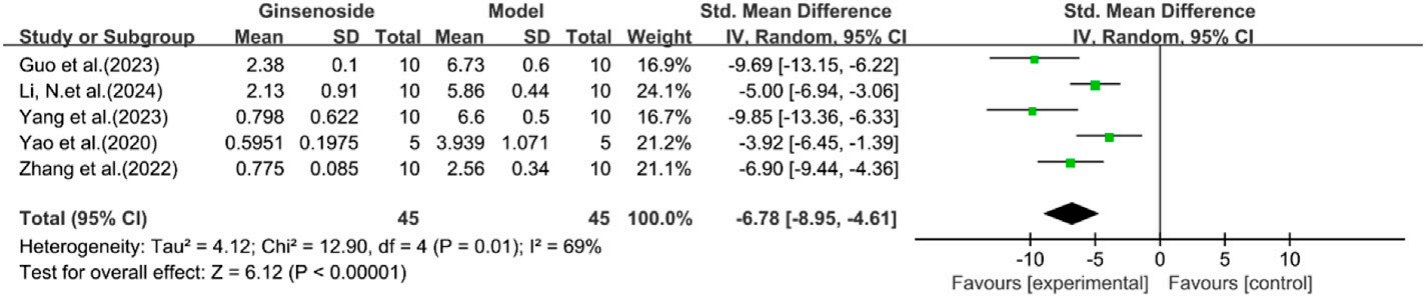
Forest plot: effect of ginsenosides on NAS score.

A total of five studies reported the effect of ginsenosides on NAS score. The pooled results showed that compared with the model group, ginsenoside group significantly reduced NAS score [n = 90, SMD = −6.78 [-8.95, −4.61], *P* < 0.05; heterogeneity: I^2^ = 69%, *P* < 0.00001] (Figure 7). Due to the limited research data, more studies are needed on the results related to histology or inflammatory markers to interpret and evaluate these results more accurately.

### Analysis of liver function

**FIGURE 8:**
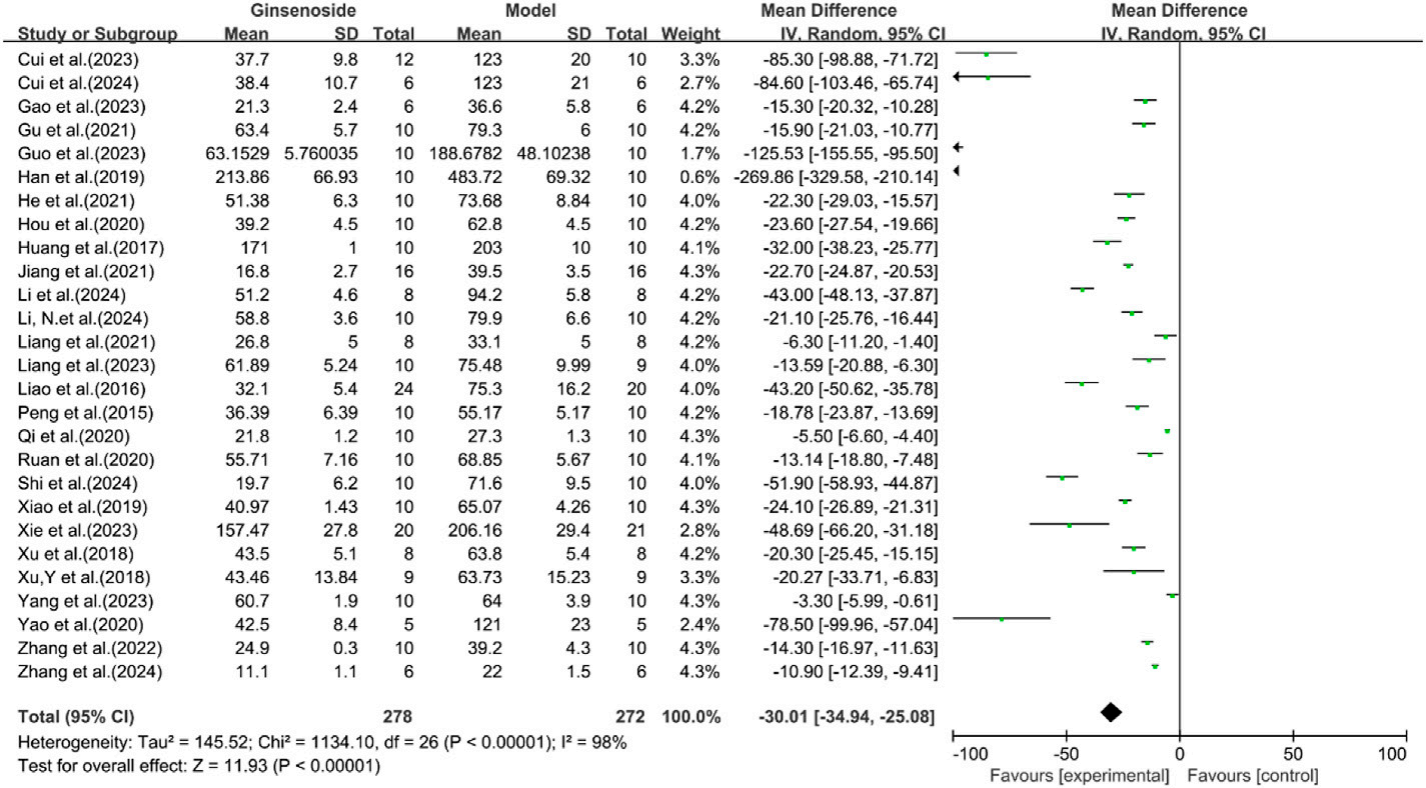
Forest plot: effect of ginsenosides on ALT level.

A total of 27 studies reported the effect of ginsenosides on ALT. The pooled results showed that compared with the model group, ginsenoside group significantly reduced ALT levels [n = 550, MD = −30.01 [-34.94, −25.08], *P* < 0.05; heterogeneity: I^2^ = 98%, *P* < 0.00001] (Figure 8).

**FIGURE 9:**
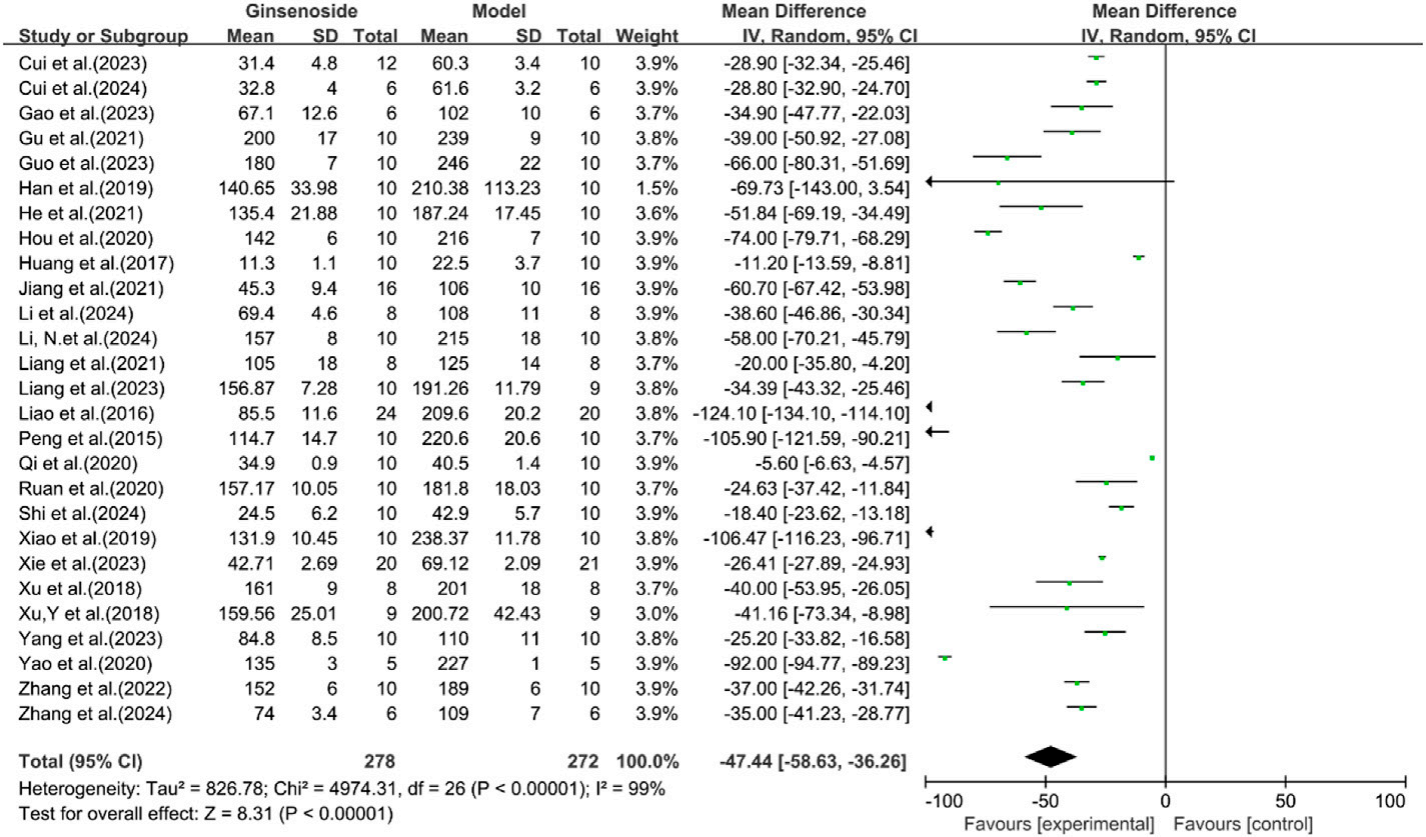
Forest plot: effect of ginsenosides on AST level.

A total of 27 studies reported the effect of ginsenosides on AST. The pooled results showed that compared with the model group, ginsenoside group significantly reduced AST levels [n = 550, MD = −47.44 [-58.63, −36.26], *P* < 0.05; heterogeneity: I^2^ = 99%, *P* < 0.00001] (Figure 9).

**FIGURE 10:**
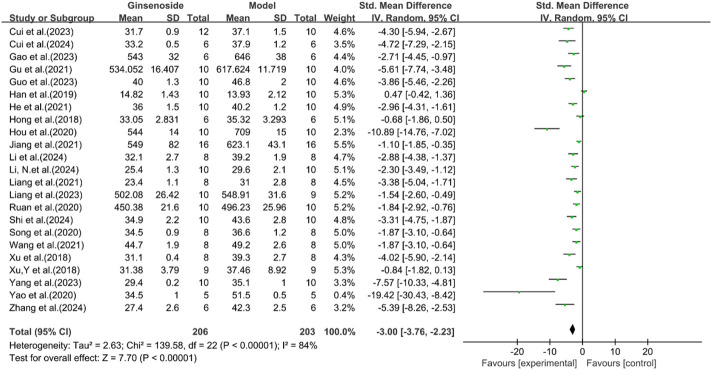
Forest plot: effect of ginsenosides on body weight level.

A total of 23 studies reported the effect of ginsenosides on body weight. The pooled results showed that compared with the model group, ginsenoside group significantly reduced body weight [n = 409, SMD = −3.00 [-3.76, −2.23], *P* < 0.05; heterogeneity: I^2^ = 84%, *P* < 0.00001] (Figure 10).

**FIGURE 11:**
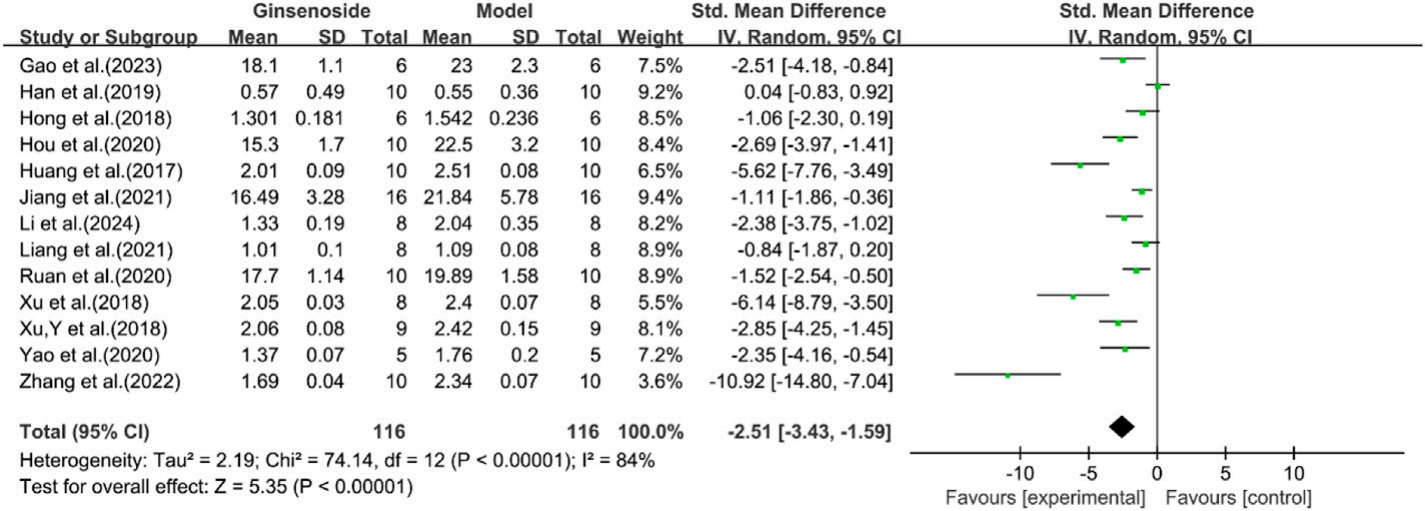
Forest plot: effect of ginsenosides on liver weight level.

A total of 13 studies reported the effect of ginsenosides on liver weight. The pooled results showed that compared with the model group, ginsenoside group significantly reduced liver weight [n = 232, SMD = −2.51 [-3.43, −1.59], *P* < 0.05; heterogeneity: I^2^ = 84%, *P* < 0.00001] (Figure 11).

**FIGURE 12:**
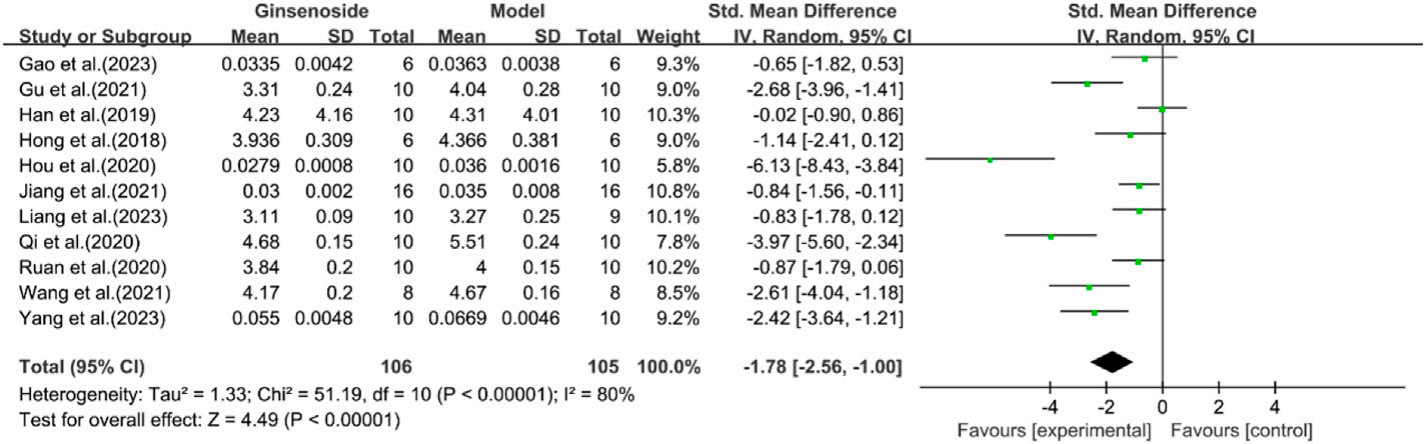
Forest plot: effect of ginsenosides on liver index level.

A total of 11 studies reported the effect of ginsenosides on liver index. The pooled results showed that compared with the model group, ginsenoside group significantly reduced liver index [n = 211, SMD = −1.78 [-2.56, −1.00], *P* < 0.05; heterogeneity: I^2^ = 80%, *P* < 0.00001] (Figure 12).

**FIGURE 13:**
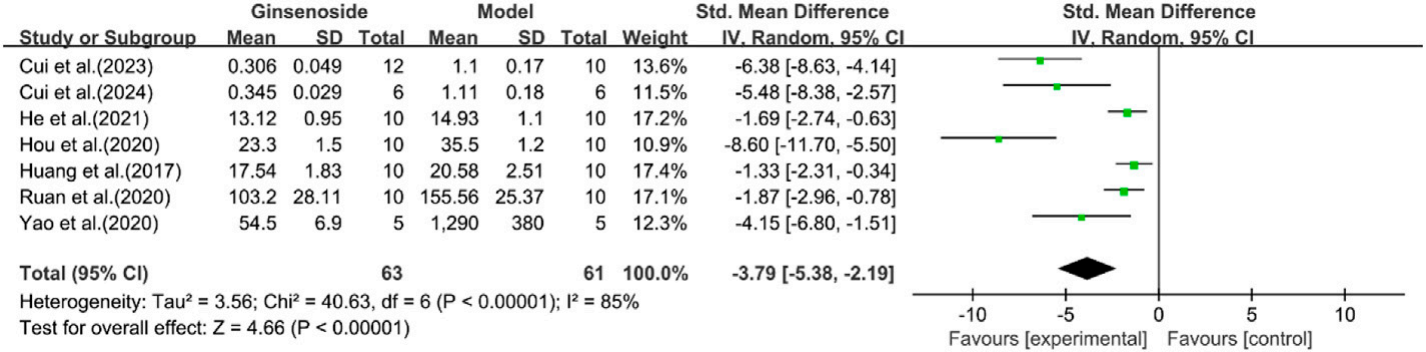
Forest plot: effect of ginsenosides on serum insulin level.

A total of seven studies reported the effect of ginsenosides on serum insulin. The pooled results showed that compared with the model group, ginsenoside group significantly reduced serum insulin levels [n = 124, SMD = −3.79 [-5.38, −2.19], *P* < 0.05; heterogeneity: I^2^ = 85%, *P* < 0.00001] (Figure 13). Except for HDL, other indicators have been reduced by ginsenosides.

### Inflammation-related indicators

**FIGURE 14:**
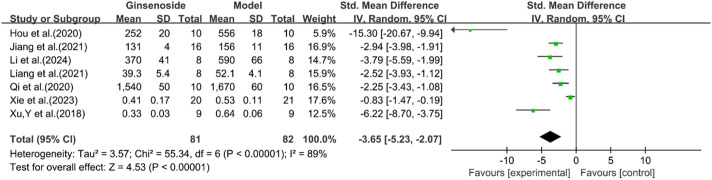
Forest plot: effect of ginsenosides on IL-1 level.

A total of seven studies reported the effect of ginsenosides on IL-1. The pooled results showed that compared with the model group, ginsenoside group significantly reduced the expression level of IL-1 [n = 163, SMD = −3.65 [-5.23, −2.07], *P* < 0.05; heterogeneity: I^2^ = 89%, *P* < 0.00001] (Figure 14).

**FIGURE 15:**
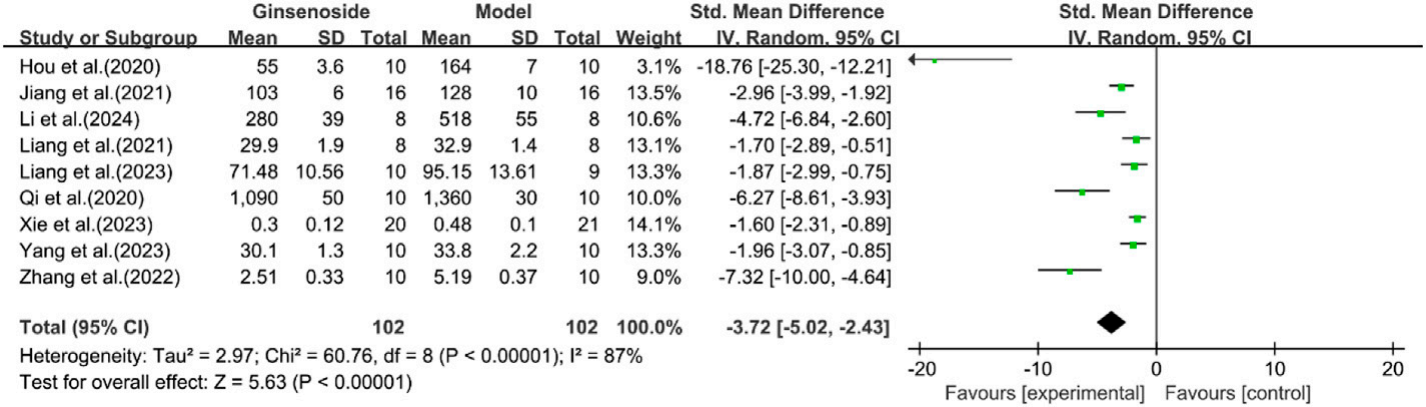
Forest plot: effect of ginsenosides on IL-6 level.

A total of eight studies reported the effect of ginsenosides on IL-6. The pooled results showed that compared with the model group, ginsenoside group significantly reduced the expression level of IL-6 [n = 204, SMD = −3.72 [-5.02, −2.43], *P* < 0.05; heterogeneity: I^2^ = 87%, *P* < 0.00001] (Figure 15).

**FIGURE 16:**
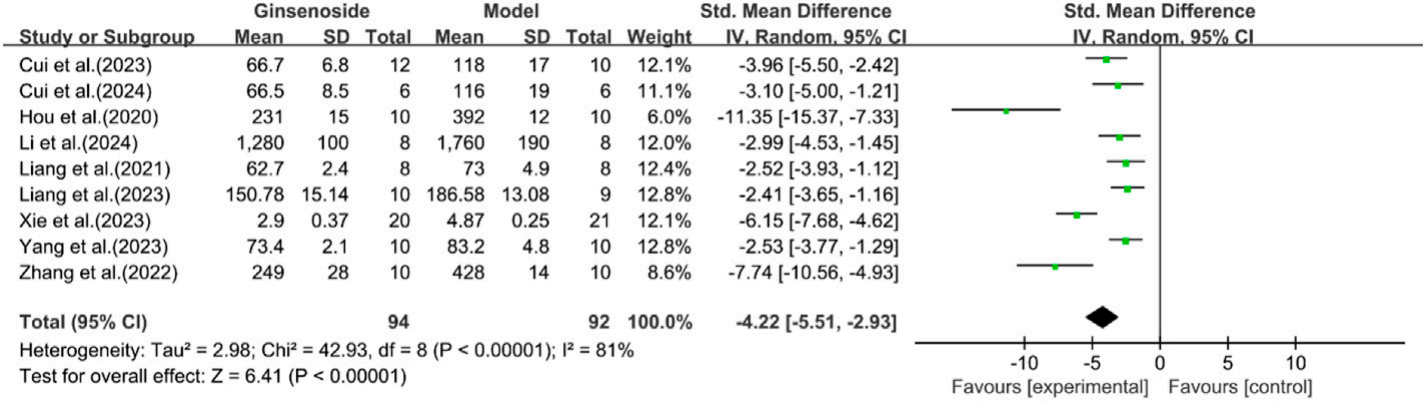
Forest plot: effect of ginsenosides on TNF-α level.

A total of nine studies reported the effect of ginsenosides on TNF-α. The pooled results showed that compared with the model group, ginsenoside group significantly reduced the expression level of TNF-α (n = 186, SMD = −4.22 [-5.51, −2.93], *P* < 0.05; heterogeneity: I^2^ = 81%, *P* < 0.00001) (Figure 16).

### Publication bias

**FIGURE 17:**
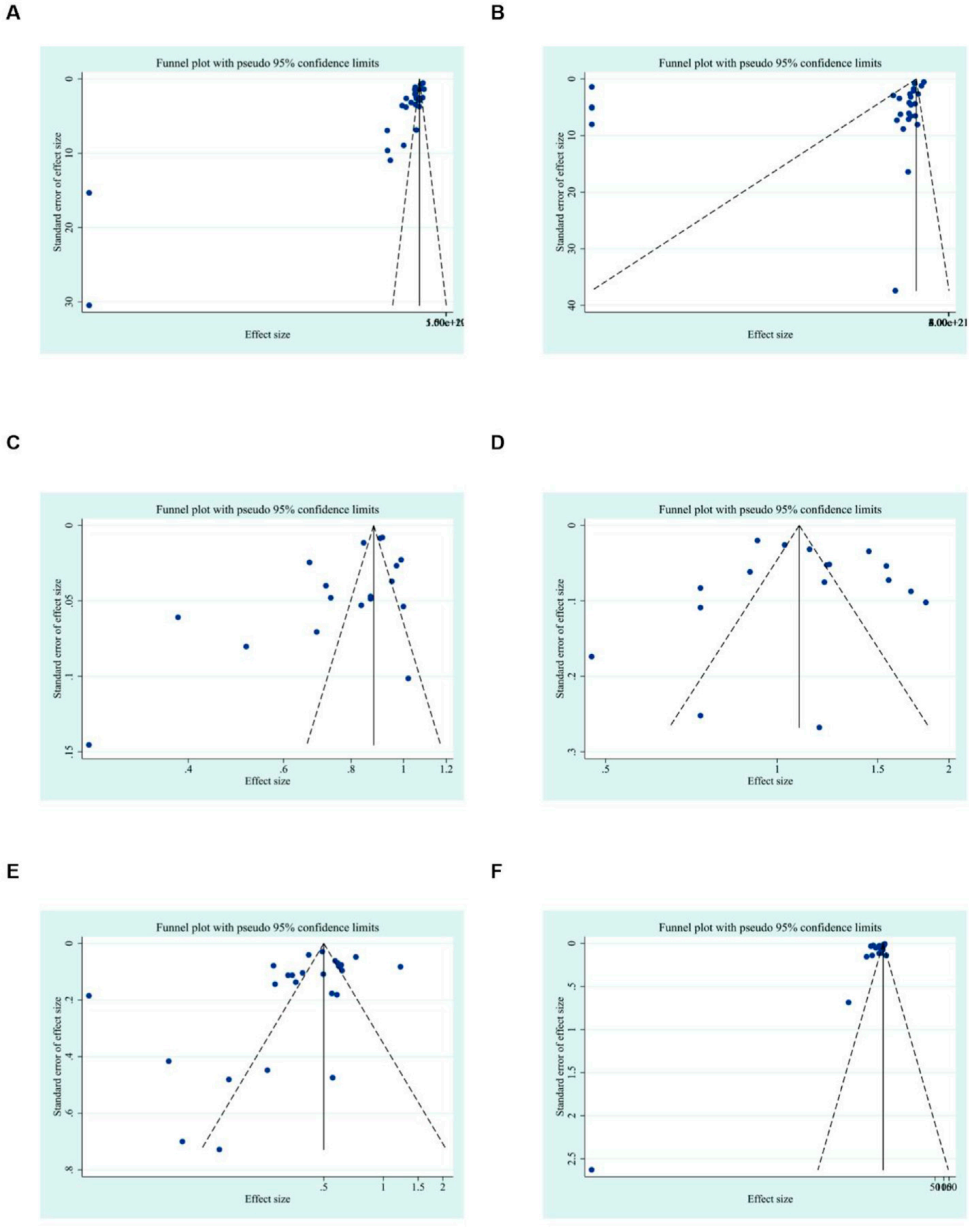
Results of funnel plots. **(A)** ALT, **(B)** AST, **(C)** LDL, **(D)** HDL, **(E)** TC, **(F)** TG.

**FIGURE 18:**
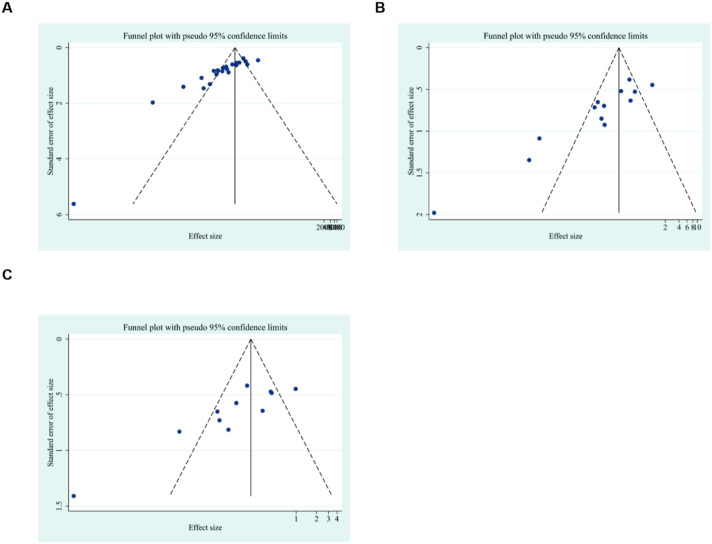
Results of funnel plots. **(A)** body weight, **(B)** liver weight, **(C)** liver index.

**FIGURE 19:**
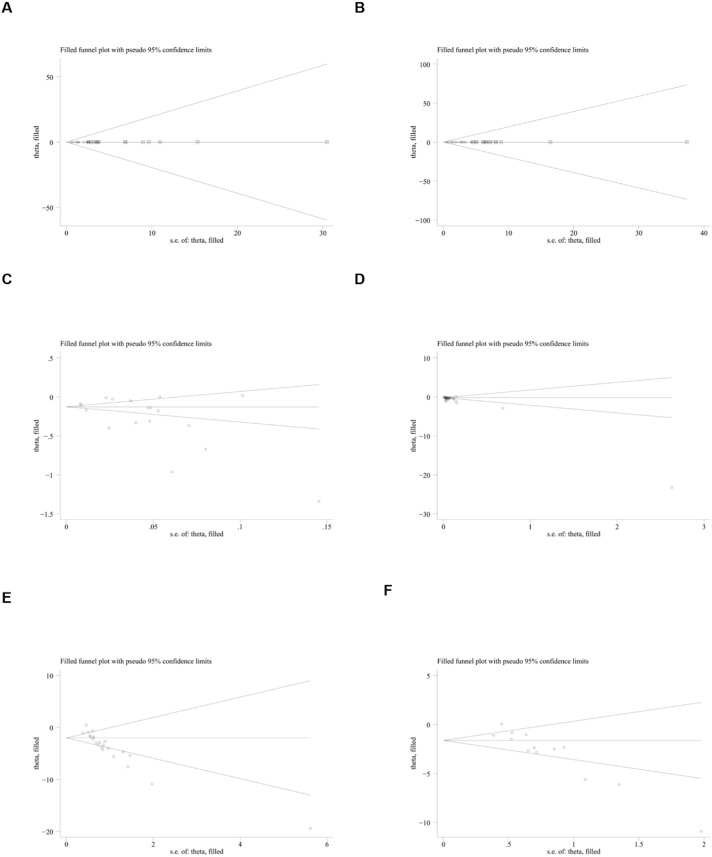
Results of trim and fll method. **(A)** ALT, **(B)** AST, **(C)** LDL, **(D)** TG, **(E)** body weight, **(F)** liver weight.

**FIGURE 20:**
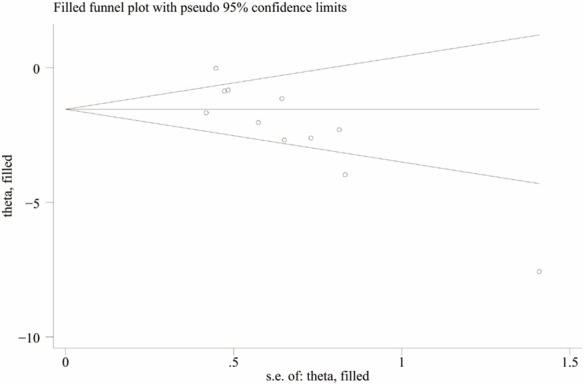
Results of liver index by trim and fll method.

To further explore publication bias, for each outcome with a minimum of 10 studies, funnel plots and Egger’s test were employed to evaluate any possible systematic biases in the publication of research findings. The results indicated the presence of publication bias for the relevant indicators, as shown in Figures 17, 18 and Supplementary Figures 2, 3. Subsequently, the trim-and-fill method was utilized to assess the influence of this publication bias on the overall results. Based on the random effects model, the results of the trim-and-fill method show that these missing study data do not affect the stability of the results (Figures 19, 20).

### Subgroup and sensitivity analysis

**FIGURE 21:**
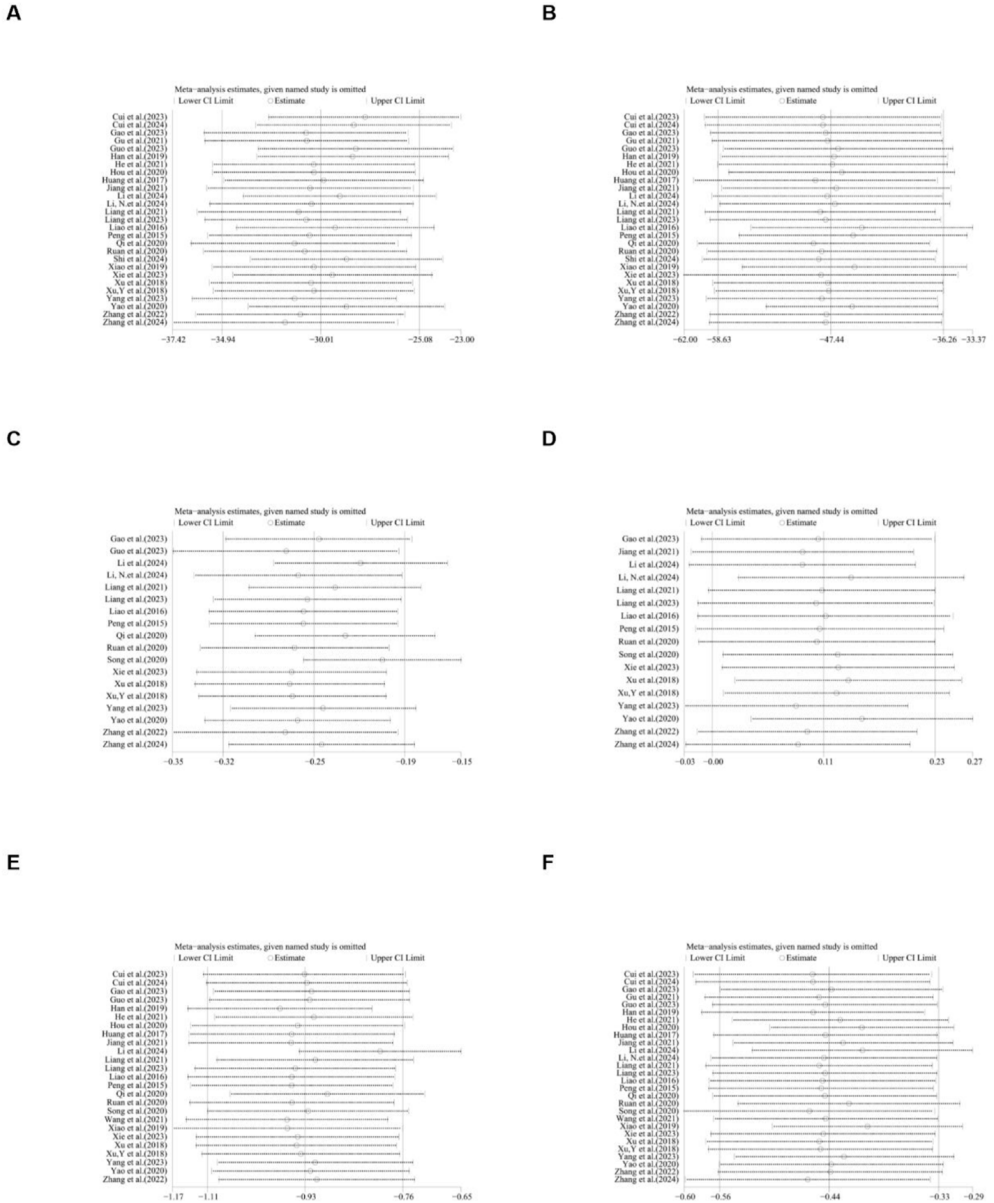
Results of Sensitivity analysis. **(A)** ALT, **(B)** AST, **(C)** LDL, **(D)** HDL, **(E)** TC, **(F)** TG.

**FIGURE 22:**
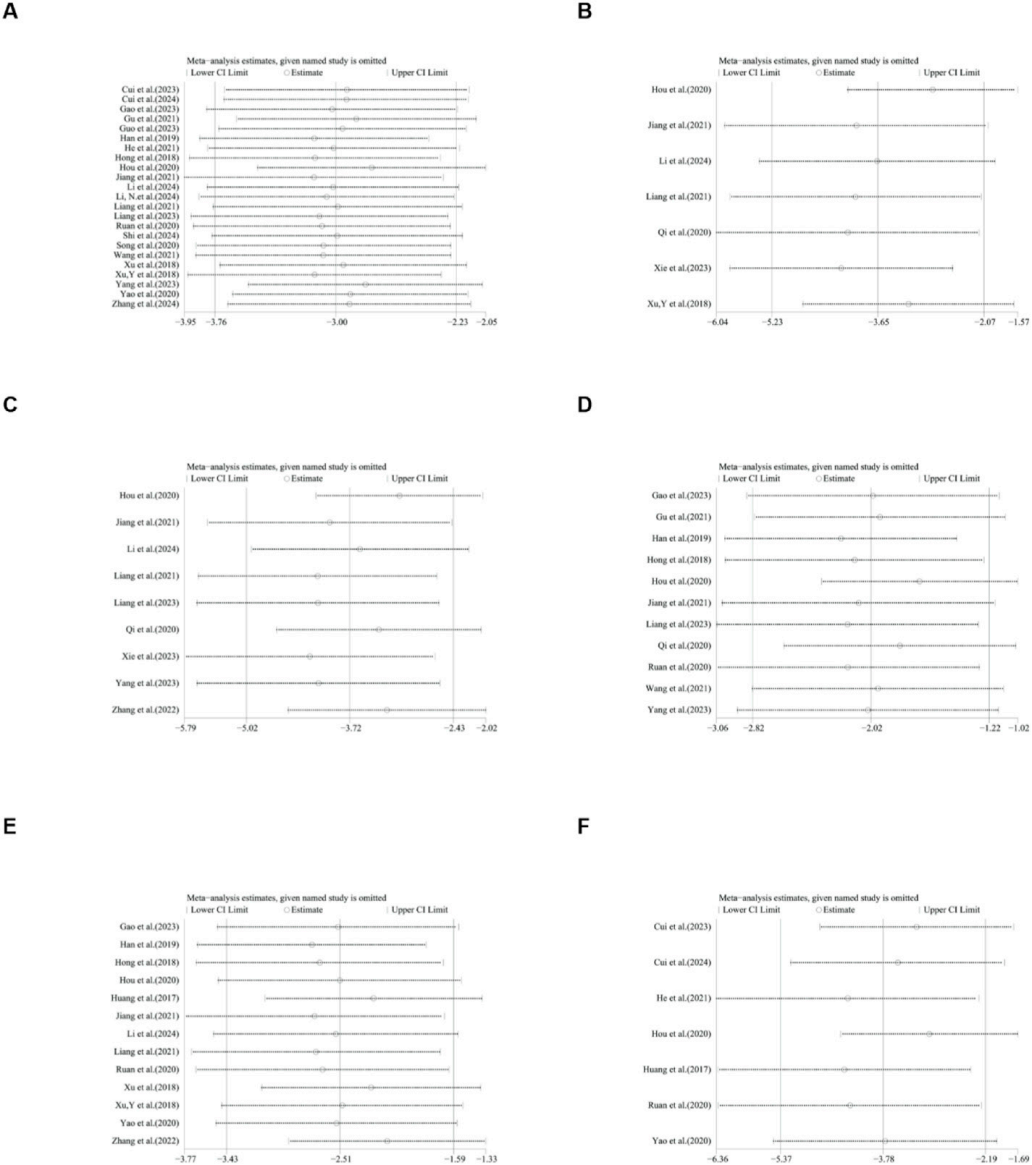
Results of Sensitivity analysis. **(A)** body weight, **(B)** IL-1, **(C)** IL-6, **(D)** liver index, **(E)** liver weight, **(F)** serum insulin.

**FIGURE 23:**
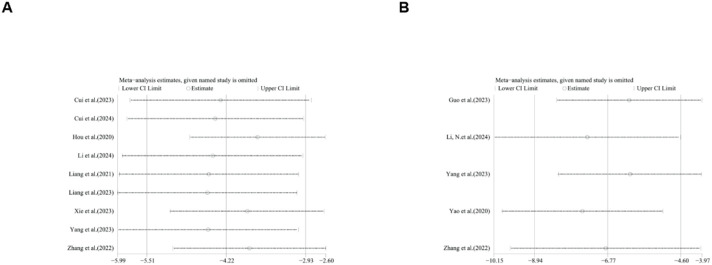
Results of Sensitivity analysis. **(A)**. TNF-α, **(B)**. NAS score.

Due to the high heterogeneity, we conducted subgroup analyses of the main results ALT, AST, TC and TG by animal strains, modeling methods, administration methods, ginsenoside dosages and types of ginsenosides. The results showed that the heterogeneity of TC may be derived from differences in modeling methods (Supplementary Table 2). For ALT, AST, HDL, LDL, TC, TG, body weight, IL-1, IL-6, liver index, liver weight, serum insulin, TNF-α and NAS score sensitivity analysis was performed by removing one study at each stage. The results show that removing one study has no significant impact on the size of the overall effect. The results are shown in Figures 21–23.

## Discussion

Our research conducted a quantitative assessment of the pharmacological effects of ginsenosides in preclinical animal models of NAFLD. A total of 30 papers were included in this research, involving a total of 604 animals. In this study, we found that ginsenosides can reduce lipid-related indicators including TC, TG, and LDL, while having no significant effect on HDL. Moreover, ginsenosides play a protective role in liver function. They decrease the levels of ALT and AST. Additionally, ginsenosides have an inhibitory effect on inflammatory factors. The levels of IL-1, IL-6 and TNF-α were found to be reduced after ginsenoside treatment. They led to a decrease in body weight, liver weight, liver index and serum insulin. Due to the high heterogeneity, we conducted subgroup analyses of the main results ALT, AST, TC and TG by animal strains, modeling methods, administration methods, ginsenoside dosages and types of ginsenosides. The results showed that the heterogeneity of TC may be derived from differences in modeling methods. Sensitivity analysis was performed by removing one study at each stage. The results show that removing one study has no significant impact on the size of the overall effect.

In this review, we conducted a meta-analysis of preclinical trials of ginsenosides in the treatment of NAFLD. The purpose is to evaluate the lipid-lowering effect and antioxidant and anti-inflammatory properties of ginsenosides in the treatment of NAFLD. Current evidence shows that ginsenosides can effectively control the progression of NAFLD through antioxidant and anti-inflammatory mechanisms. This meta-analysis is based on animal experiments, so it cannot represent the results of clinical trials, but it still has certain reference value and guiding significance for future experiments.

The clinical transformation strategies of ginsenosides: Although this study confirmed the significant efficacy of ginsenosides on NAFLD in animal models, translating them into clinical applications still faces key challenges in bioavailability. Bioavailability is an obstacle to the conversion of ginsenosides for human use, which is quite appropriate, as many ginsenosides have low oral bioavailability or require intestinal microbiota conversion (such as in the case of compound K). Interestingly, the new drug delivery system may offer better solubility, oral absorption rate and bioavailability. A series of nano-delivery systems have been developed to enhance efficiency and reduce related adverse reactions (Kim et al., 2018). It is worth noting that the ginsenoside compound K prepared with polymer micelles has good biodegradability and biocompatibility, and its anti-tumor effect is stronger than that of free compound K (Yang et al., 2017). In order to enhance the bioavailability level, Rb1 has been encapsulated into nano-RB1 using polymer nano-capsules, thereby significantly inhibiting the activities of NF-κB and NLRP3 inflammasomes (Liu et al., 2020). However, as far as we know, although progress has been made in the development of traditional Chinese medicine, there is still no compound K preparation available for patients. Currently, compound K capsules are undergoing clinical trials for the treatment of rheumatoid arthritis (Chen L. et al., 2017).

Comparison of other intervention measures: In this study, ginsenoside intervention demonstrated good therapeutic potential. Compared with other intervention methods, the effect of ginsenosides is unique. Dietary management is a very promising strategy for intervening in NAFLD. panaxadiol saponin component (PDS-C) can significantly alleviate the liver function, liver steatosis and blood lipid levels of mice with NAFLD, and reduce oxidative stress and inflammation. Supplementation of PDS-C reduces insulin resistance and glucose homeostasis in mice with NAFLD, although its therapeutic effect is not as obvious as that of metformin (Mi et al., 2024). Compound K and metformin reduces glucose intolerance and hepatic steatosis in rats fed with HFD through AMPK activation (Hwang et al., 2018).

Comparison with relevant literature: A meta-analysis by Yang et al. (2023a) examined the efficacy of ginseng in animal studies of NAFLD (including 41 studies). Although this study focused on the overall formulation of ginseng (rather than just isolated ginsenosides), it reported similar significant reductions in lipid parameters and transaminases after treatment with ginseng. And HDL has also significantly increased (Yang K. et al., 2023). This study quantitatively evaluated the efficacy of ginsenosides in NAFLD, complementing the meta-analysis of 41 studies included by Yang et al. (2023b). This study indicates that the whole ginseng preparation can significantly increase HDL levels, while no similar effect was observed in this study. This difference may stem from the complexity of the components, while purified ginsenosides lack such synergistic effects. In addition, Yang et al.‘s research covered different ginseng varieties and administration routes, while this study focused on a single purified component, more clearly revealing the direct mechanism of action of ginsenosides. This suggests that future studies can explore the strategy of “combined administration of ginsenosides and non-ginsenosides components” to integrate the advantages of both and provide a more comprehensive treatment plan for NAFLD.

However, this study has some inevitable limitations. First, some studies did not provide detailed baseline characteristics. At the same time, given the insufficient methodological quality of some studies, the results of this study should be interpreted with caution, and more qualitative studies are needed in the future. The absence of such baseline data makes it more difficult to confirm whether each group is comparable before treatment, although these studies usually use the same species of animals. Second, there is publication bias in this study, which may be related to the quality of the included literature. Animal experiments that produce negative results contrary to its hypothesis are less likely to be reported. More data (and perhaps more consistent experimental designs among studies) are needed to accurately identify the sources of heterogeneity. Due to the high heterogeneity, we conducted subgroup analyses of the main results ALT, AST, TC and TG by animal strains, modeling methods, administration methods, ginsenoside dosages and types of ginsenosides. Only the heterogeneity results of TC suggest that it may stem from the differences in modeling methods. Moreover, the data in some studies are not provided directly as raw data but are obtained indirectly through data extraction tools, which may lead to data measurement bias. In addition, many of the included studies conducted hematoxylin-eosin (H&E) staining tests on liver histology, but only a few of the five studies reported quantitative histological results that could be used for combined analysis. Therefore, this meta-analysis mainly focused on biochemical results. While the amelioration of hepatic enzyme levels and normalization of lipid profiles suggest clinical benefits in NAFLD management, conclusive quantitative validation of steatosis regression or fibrosis attenuation remains insufficiently characterized through current diagnostic modalities. Furthermore, due to the relatively small sample size of the research and the certain complexity of the research data. Multiple comparisons significantly increase the probability of Type I error (false positive error). Moreover, excessive comparisons reduce the statistical test power, increasing the risk of Type II error (false negative error) and potentially overlooking real differences. The complexity of multiple comparison results also complicates data analysis and conclusion drawing, interfering with the accurate determination of the true relationships among various factors. In this study, sensitivity analysis based on study quality was not conducted due to several constraints. First, the included studies exhibited high data heterogeneity, with significant variations in study subject characteristics, interventions, and measurement methods. Second, data missing was prevalent, rendering a reliable quality assessment system unfeasible. Third, the absence of a universally accepted quality evaluation standard in the field, along with varying assessment tools, precluded consistent quality grading. Finally, the diversity in study designs, such as grouping, sample size, and observation period, reduced comparability among studies, making it difficult to isolate the impact of quality on results. Collectively, these factors rendered sensitivity analysis based on study quality infeasible in this research. Moreover, the lack of key information in some studies led to significant uncertainties in the quality assessment. There is minimal evidence of the efficacy of ginsenosides in the treatment of NAFLD *in vitro* and *in vivo* experiments. Therefore, clinical examinations are needed. However, due to concerns about bioavailability, the transition from preclinical to clinical research has been delayed.

### The protective effect of ginsenosides in NAFLD

**FIGURE 24:**
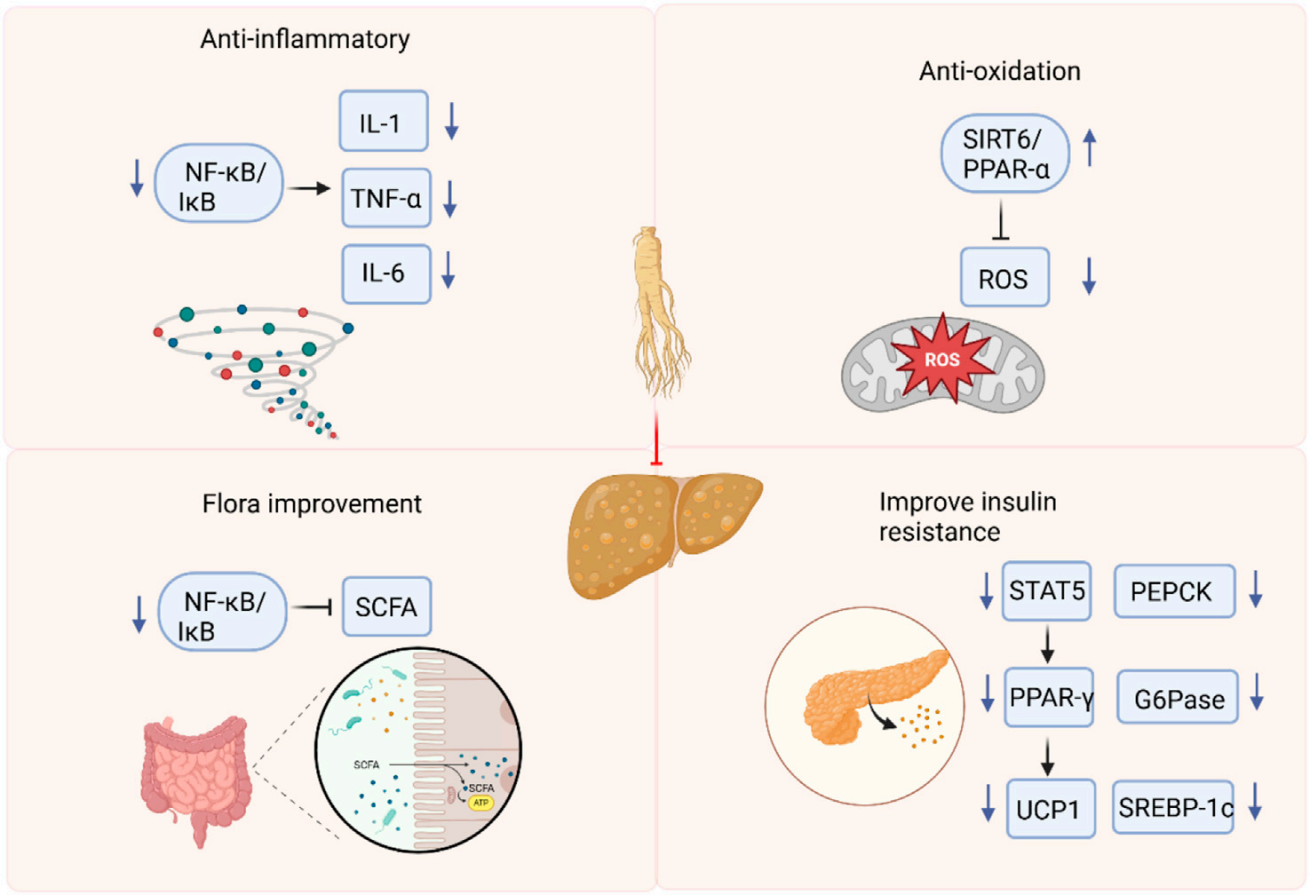
Role and mechanisms of ginsenoside in treating NAFLD.

Based on the findings of this review, ginsenosides have demonstrated efficacy across a diverse range of NAFLD and NASH models. Through a comprehensive meta-analysis of the included studies, we have constructed a mechanistic diagram (Figure 24).

#### Anti-inflammation and antioxidation

In the absence of other chronic liver diseases and alcoholism, NAFLD is characterized by a fat accumulation in the liver that exceeds 5%. NAFLD ranges from simple steatosis to NASH, which is characterized by liver lesions, inflammation, and fibrosis (Cobbina and Akhlaghi, 2017). Elevated reactive oxidative stress, lipid peroxidation, inflammation, and fibrosis lead to tissue damage and play an important role in the development and progression of NAFLD (Pereira et al., 2022). Nuclear factor-κB (NF-κB) is the main regulator of the inflammatory response and shows an antagonistic relationship in controlling inflammation (de Gregorio et al., 2020). Ginsenoside Rg1 has been shown to suppress the activity of NF-κB signaling pathway, thereby preventing the production of pro-inflammatory cytokines. The activation of NOD-like receptor protein 3 (NLRP3) inflammasome occurs in NAFLD. NLRP3 inflammasome blockade can alleviate liver inflammation and fibrosis in experimental NASH in mice (Mridha et al., 2017). Specifically, Rg1 has been shown to inhibit the activation of the NLRP3 inflammasome, leading to a significant reduction in the production of pro-inflammatory cytokines, such as IL-1β and IL-18 (Xu YS. et al., 2018). Moreover, Rg1 maintains the activity of forkhead box O1 (FOXO1) in the liver, which not only enhances its antioxidant capacity but also suppresses inflammation and promotes metabolic homeostasis. Rg1 attenuates NAFLD progression in D-galactose-induced murine models through targeted hepatoprotection, effectively mitigating steatotic accumulation, inflammatory cascades, and hepatocellular damage (Qi et al., 2020). Rg1 reduces ALT/AST levels and pro-inflammatory cytokines, and effectively reduces hepatic steatosis and inflammation, which may be related to the AMP-activated protein kinase (AMPK)/NF-κB pathway (Xiao Q. et al., 2019). Mitochondria are the primary source of reactive oxygen species (ROS) in cells. The intimate connection between mitochondria and the endoplasmic reticulum (ER) *via* the mitochondria-associated membrane means that ROS generated by mitochondria can trigger ER stress (Burgos-Morón et al., 2019). Studies have shown that ginsenoside Rg1 alleviates NAFLD by inhibiting lipid peroxidation and endoplasmic reticulum stress in HFD-induced NAFLD mice. In db/db mice with NAFLD, ginsenoside Re exerts its protective effects possibly through the inhibition of oxidative stress and the upregulation of peroxisome proliferator - activated receptor γ (PPARγ) expression (Jiang et al., 2021).

#### Regulating intestinal flora

Intestinal flora imbalance plays a key role in the pathogenesis of NAFLD through its metabolites. Restoration of the intestinal microbiota and supplementation of symbiotic bacterial metabolites offer potential therapeutic strategies for this condition (Chen and Vitetta, 2020). Ginsenosides can regulate the composition and diversity of the intestinal flora and promote the metabolic homeostasis of the microbial community (Zheng et al., 2021). Metabolites of the intestinal flora, including short-chain fatty acids, tryptophan and its derivatives, play a crucial role in regulating intestinal and systemic immune homeostasis (Li et al., 2023). Ginsenoside extract (GE) exerts multiple beneficial effects, such as regulating the intestinal microbiota, enhancing the intestinal barrier function, restoring energy balance, and alleviating metabolic inflammation. In addition, GE may become a potential drug for preventing NAFLD by integrating prebiotic, anti-inflammatory and energy-regulating effects (Liang et al., 2021). Ginsenoside Rk3 treatment significantly changed the abundance of short-chain fatty acids. These changes are related to beneficial changes in the species and composition of the intestinal microbiota. Ginsenoside Rk3 helps to clarify the interaction between the host and microorganisms, positioning it as a promising candidate for the treatment of NASH (Guo et al., 2023). After Rh4 treatment, the degree of hepatic steatosis, the level of lobular inflammation and bile acids in liver tissue are reduced. At the same time, Rh4 treatment significantly increases the levels of intestinal short-chain fatty acid and bile acids, and accompanied by complementary diversity and composition of the intestinal microbiota (Yang et al., 2023a). The analysis performed by 16 S rRNA sequencing revealed that Rg5 intervention induced changes in the composition of the intestinal microbiota, promoted an increase in beneficial bacteria such as *Bacteroides* and *Akkermansia*, and at the same time reduced the relative abundance of harmful bacteria, taking *Olsenella* as an example (Shi et al., 2024).

#### Improving insulin resistance

NAFLD is intricately linked to hepatic insulin resistance. The accumulation of hepatic diglycerides activates protein kinase C - ε (PKC - ε), which in turn impairs insulin receptor activation and insulin-stimulated glycogen synthesis (Yang SM. et al., 2023). In rats fed a HFD, significant elevations are observed in liver function markers, blood lipid levels, glucose intolerance, and insulin resistance (Chen XJ. et al., 2017). Ginsenoside Mc1 exerts a protective effect on ER stress-induced apoptotic damage, insulin resistance and lipogenesis in palmitate-treated hepatocytes and livers of diet-induced obesity (DIO) mice. Mc1 may be a potential therapeutic strategy for preventing NAFLD in obese and insulin-resistant patients (Roh et al., 2020). Ginsenoside Rg3 reduces obesity-induced insulin resistance and lipotoxicity through signal transducer and activator of transcription 5 (STAT5)-PPAR-γ (Lee et al., 2017). Ginsenoside Rg1 prevents liver insulin resistance by maintaining insulin signal sensitivity and is a promising alternative drug (Mo et al., 2019). HCV core protein binding protein 6 (HCBP6) plays a role in regulating lipolysis and fatty acid oxidation. In addition, ginsenoside Rh2 upregulates the expression of HCBP6. As a result, administration of ginsenoside Rh2 reduces HFD-induced fatty liver and glucose tolerance (Lu et al., 2020).

## Conclusion

This systematic preclinical assessment demonstrates that ginsenosides effectively reduce body weight, liver weight, and other pathological manifestations associated with NAFLD. Mechanistically, ginsenosides significantly decrease lipid accumulation, alleviate insulin resistance, and inhibit inflammatory responses and oxidative stress. These findings underscore the unique therapeutic potential of ginsenosides in the treatment of NAFLD and NASH. However, future research involving more rigorously designed experimental models is essential for a more comprehensive and cautious interpretation of these conclusions.

## Data Availability

The original contributions presented in the study are included in the article/Supplementary Material, further inquiries can be directed to the corresponding authors.
