# Supplementary material for: Novel insights from meta-analysis: the efficacy of ginsenosides in non-alcoholic fatty liver disease
Source: Front Pharmacol. 2025 May 27;16:1564852. doi: 10.3389/fphar.2025.1564852 (PMC12148916; doi:10.3389/fphar.2025.1564852)

**Supplementary Figures**

Supplementary Fig.1 Risk of bias graph


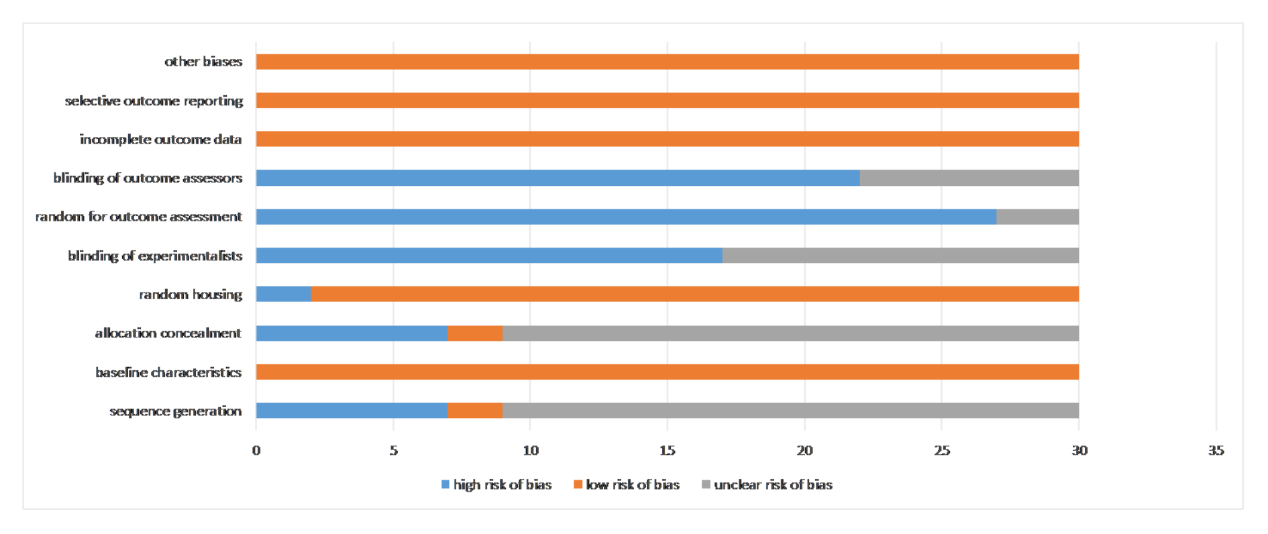


Supplementary Fig.2 The results of Egger tests A.ALT, B.AST, C.LDL, D.HDL, E.TC, F. TG


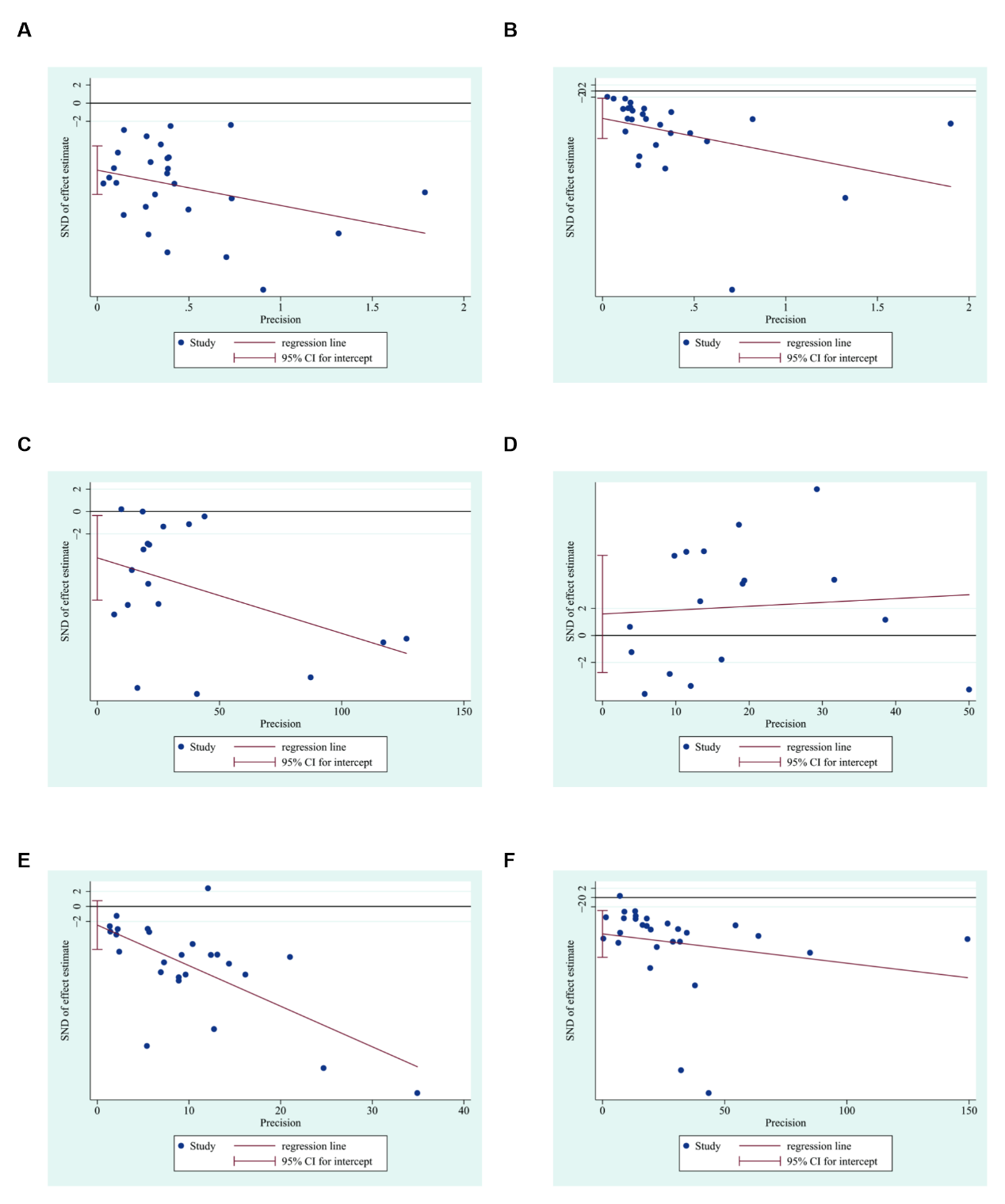


Supplementary Fig.3 The results of Egger tests A. body weight, B. liver weight, C. liver index


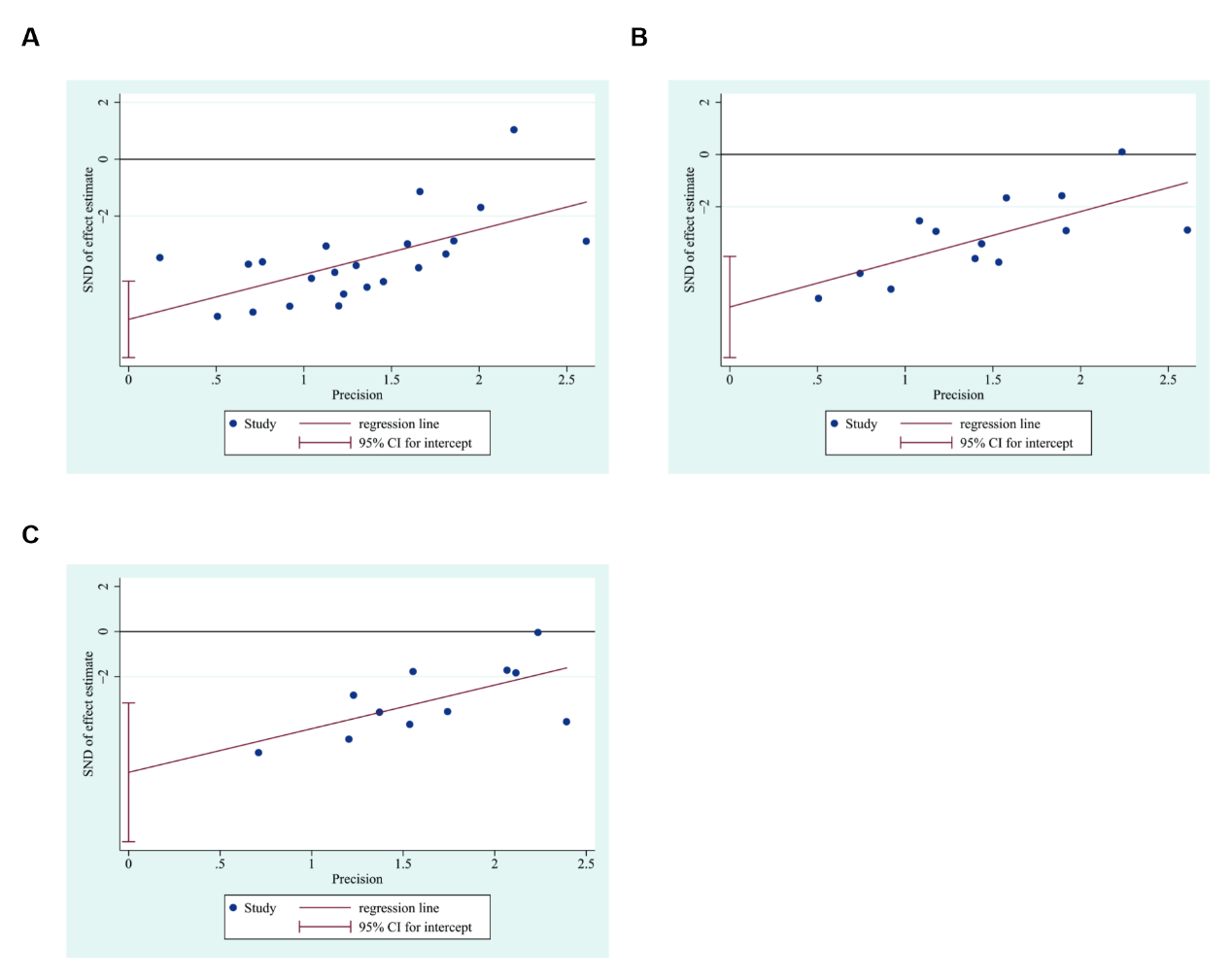

Supplement: Supplementary file 1 [file Supplementaryfile1.docx]
