# Supplementary material for: Novel insights from meta-analysis: the efficacy of ginsenosides in non-alcoholic fatty liver disease
Source: Front Pharmacol. 2025 May 27;16:1564852. doi: 10.3389/fphar.2025.1564852 (PMC12148916; doi:10.3389/fphar.2025.1564852)
Supplement: Supplementary file 2 [file Supplementaryfile2.docx]

**Supplementary Tables**

Supplementary Table 1 Characteristics of the included studies

| Study (year) | Species (sex, n = ginsenosides/ model group, age, and weight) | Model method | Ginsenosides group (administration, drug dose, duration) | Model group (administration, drug dose, duration) | Outcomes | Name of Ginsenoside | Mechanism | Effect |
| --- | --- | --- | --- | --- | --- | --- | --- | --- |
| Cui et al.(2023)(1) | C57BL/6 mice (male,12/10,6-8 weeks) | HFD | Intraperitoneal injection,5/10/15 mg/kg, 5 weeks | Normal saline | AST, ALT, TC, TG, body weight, serum insulin, TNF-α | Ginsenoside Rd | Acts as an activator of SIRT6, directly binds to SIRT6, enhances its deacetylase activity, regulates the SIRT6/PPARα signaling pathway | Reduces the body weight of mice, decreases the contents of triglyceride (TG) and total cholesterol (TC), decreases the levels of alanine transaminase (ALT) and aspartate transaminase (AST), alleviates insulin resistance and inflammatory stress |
| Cui et al.(2024)(2) | C57BL/6 mice (male,6/6, 8 weeks, 20-25g) | HFD | Intraperitoneal injection, 5/10/15mg/kg, 4 weeks | Normal saline | AST, ALT, TC, TG, body weight, serum insulin, TNF-α, IL-1 | Ginsenoside Rd | Decreases ROS levels and inflammatory levels, and increases the rate of fatty acid oxidation. | Reduces the body weight of HFD mice, increases serum ketone body levels, lowers the contents of TG and TC in the liver and serum |
| Gao et al.(2023)(3) | SD rats (male, 6/6,180-200g) | HSFD | Gavage,25/100 mg/kg,4 weeks | Normal saline | AST, ALT, TC, TG, HDL, LDL, body weight, liver weight, liver index | Ginsenoside Rg1 | Alleviates liver steatosis and oxidative stress | Decreases the expression of serum TG, TC, ALT, AST, LDL-C |
| Gu et al.(2021)(4) | SD rats (male, 10/10,6 weeks, 180-220 g) | HSFD | Gavage,100 mg/kg/d, 8 weeks | Normal saline | AST, ALT, TC, body weight, liver index | Ginsenoside Rg1 | Regulates key genes Atf3 and Acox2 | Protects liver function and alleviates the pathological process of NAFLD |
| Guo et al.(2023)(5) | C57BL/6 mice (male,10/10,6-8 weeks,20-25 g) | HSFD and CCl4 | Gavage,60/120 mg/kg, 4 weeks | Tea oil | AST, ALT, TC, TG, LDL, body weight, NAS score | Ginsenoside Rk3 | Significantly inhibits the PI3K/AKT signaling pathway | Reduces the liver inflammation, lipid deposition, and fibrosis in mice caused by the above modeling methods. It also remarkably changes the abundance of short-chain fatty acids and brings about beneficial changes in the variety and composition of the intestinal microbiota |
| Han et al.(2019)(6) | C57BL/6 mice (male,10/10,6 weeks,20-25 g) | HFD | Gavage,40/80 mg/kg,15 days | No mention | AST, ALT, TC, TG, body weight, liver weight, liver index | Ginsenoside Rb1 | Reduces lipid peroxidation | Reduces plasma ALT, AST, TC and TG |
| He et al.(2021)(7) | C57BL/6 mice (male,10/10,8 weeks,16-20 g) | HFD | Gavage,20/40 mg/kg,12 weeks | Normal saline | AST, ALT, TC, TG, body weight, serum insulin | Ginsenoside Rg1 | Inhibits of the TGF-β1/p-Smad3/Smad pathway and the Wnt/β-catenin pathway | Reduces body weight, insulin resistance, and serum glucose, insulin |
| Hong et al.(2018)(8) | C57BL/6 mice (male,6/6,6 weeks) | HFD | Intraperitoneal injection, 40 mg/kg, 10 days | PBS | body weight, liver weight, liver index | Ginsenoside Rb2 | Increases the expression of PPARα | Reduces liver weight, alleviates liver steatosis |
| Hou et al.(2020)(9) | SD rats (male,10/10,180-220g) | HSFD | Gavage,30/60 mg/kg/d,4/8 weeks | No mention | AST, ALT, TC, TG, body weight, liver weight, liver index, serum insulin, IL-1, IL-6, TNF-α | Ginsenoside Rg1 | Inhibits the inflammatory response, regulates lipid metabolism | Reduces the levels of INS, TG, and TC, protects liver function, and and inhibits lipid accumulation in the liver |
| Huang et al.(2017)(10) | db/db mice (male,10/10,6 weeks) | HSFD | Intraperitoneal injection,10 mg/kg/d,4 weeks | Normal saline | AST, ALT, TC, TG, liver weight, serum insulin | Ginsenoside Rb2 | Alleviates hepatic lipid accumulation, and reduces glucose tolerance by restoring autophagy through inducing sirt1 and activating AMPK | Impairs glucose tolerance, decreases hepatic lipid accumulation, and restores hepatic autophagy in male db/db mice |
| Jiang et al.(2021)(11) | SD rats (male,16/16,6 weeks,180-200g) | HSFD | Gavage,25/50/100 mg/kg,8 weeks | No mention | AST, ALT, TC, TG, HDL, body weight, liver weight, liver index, IL-1, IL-6 | Ginsenoside Rg1 | Affects the TGF-β1/Smad signaling pathway | Reduces AST,ALT,TC and TG |
| Li et al.(2024)(12) | C57BL/6 mice (male,8/8,4-6 weeks) | HFD | Intraperitoneal injection,1 mg/kg, 8 weeks | Normal saline | AST, ALT, TG, TC, HDL, LDL, body weight, liver weight | Ginsenoside Rk3 | Regulates the PPARγ/miR-103-3p pathway | Alleviates NAFLD-induced decreases in liver function, reverses NAFLD-mediated pathological injury in liver tissue, reduces hepatic lipid accumulation and inflammatory lesions in mice |
| Li, N.et al.(2024)(13) | C57BL/6 mice (male,10/10) | HSFD and CCl4 | Gavage,60/120 mg/kg, 4 weeks | 0.5% CMC-Na | AST, ALT, TC, TG, HDL, LDL, NAS score | Ginsenoside Rg5 | Impedes the progression of NASH by regulating the Notch1 signaling pathway | Inhibits hepatic lipid accumulation and oxidative stress, reduces hepatocyte apoptosis, suppresses the presence of fibrotic proteins in the liver, and decreases the expression levels of Notch1 and its ligand Jagged-1 |
| Liang et al.(2021)(14) | C57BL/6 mice (male,8/8,6 weeks) | HFD | Gavage,100/200 mg/kg, 12 weeks | No mention | AST, ALT, TC, TG, HDL, LDL, body weight, liver weight, IL-1, IL-6, TNF-α | Ginsenosides | Inhibits NF-κB/IκB signaling activation | Regulates the gut microbiota and enhances the gut barrier function, |
| Liang et al.(2023)(15) | SD rats (male,10/9,180-220g) | HFD | Gavage,10 mg/kg,4 weeks | Normal saline | AST, ALT, TC, TG, HDL, LDL, body weight, liver index, IL-6, TNF-α | Ginsenoside Rb1 | Regulating transcription, cell proliferation, and inflammatory responses through 134 potential targets | Reduces the levels of liver coefficient, serum AST, ALT, TC, TG, LDL-C, and increases HDL-C level |
| Liao et al.(2016)(16) | SD rats (male,24/20,160-180g) | HSFD | Gavage,5,10,20 mg/kg,8 weeks | No mention | AST, ALT, TC, TG, HDL, LDL | Ginsenoside Rg1 | Regulates β-oxidation | Reduces AST, ALT, TC, TG, LDL-C, and increases HDL-C level |
| Peng et al.(2015)(17) | SD rats (male,10/10,160-180g) | HFD | Gavage,5,10,20 mg/kg,8 weeks | Normal saline | AST, ALT, TC, TG, HDL, LDL | Ginsenoside Rg1 | Regulates β-oxidation | Reduces AST, ALT, TC, TG, LDL-C |
| Qi et al.(2020)(18) | C57BL/6 mice (male, 10/10,6-8 weeks,14-18 g) | HFD | Intraperitoneal injection,40 mg/kg/d,42 days | Normal saline | AST, ALT, IL-1β, IL-6, liver index | Ginsenoside Rg1 | Maintains FOXO1 activity in the liver | Reduces ALT and AST in serum, hepatic steatosis |
| Ruan et al.(2020)(19) | Wistar rats (male,10/10,115-145g) | HSFD | Gavage,100 mg/kg,4 weeks | Normal saline | AST, ALT, TC, TG, HDL, LDL, body weight, liver weight, liver index, serum insulin | Ginsenosides | Regulates bile acid metabolism and insulin resistance | Reduces AST, ALT, TC, TG |
| Shi et al.(2024)(20) | C57BL/6 mice (male,10/10,8 weeks,18-22g) | HFD | Gavage,50/100 mg/kg, 12 weeks | Normal water | AST, ALT, TC, TG, LDL, body weight | Ginsenoside Rg5 | Activates the LKB1/AMPK/mTOR signaling pathway | Decreases expression of oxidative stress and liver injury markers |
| Song et al.(2020)(21) | C57BL/6 mice (male,8/8,7 weeks) | HFD | Gavage,10 mg/kg, 8 weeks | Normal saline | TC, TG, HDL, LDL, body weights | Ginsenoside Rb1 | Alleviates HFD - induced apoptosis of hepatocytes in mice by acting through PPAR - γ | Elevates the hepatic PPAR - γ level and suppresses hepatocytic apoptosis |
| Wang et al.(2021)(22) | C57BL/6 mice (male,8/8,6 weeks) | HFD | Intraperitoneal injection,40 mg/kg,4 weeks | Normal saline | TC, TG, body weight, liver index | Ginsenoside Rb2 | Regulates the SIRT6/PGC-1α/PTP1B pathway | Reduces body weight, liver weight/body weight ratio, and serum TC and TG |
| Xiao et al.(2019)(23) | SD rats (male,10/10,180-200g) | HSFD | Gavage,30/60 mg/kg,8 weeks | No mention | AST, ALT, TC, TG | Ginsenoside Rg1 | Regulates related metabolic pathways | Reduces blood lipid levels. In terms of liver function, the levels of ALT and AST |
| Xie et al.(2023)(24) | SD rats (male,20/21,180-220g) | HSFD | Gavage,20/40 mg/kg,8 weeks | Normal saline | AST, ALT, TC, TG, HDL, LDL, IL-1, IL-6, TNF-α | Ginsenoside Rg1 | Inhibits hepatocyte apoptosis, promotes fat breakdown, and inhibits inflammatory responses | Reduces AST, ALT, TG, TC, LDL-C, TNF-α, IL-6, and IL-1β |
| Xu et al.(2018)(25) | C57BL/6 mice (female,8/8,6-8 weeks) | HFD | Gavage,20/40 mg/kg, 1 month | 0.9% saline | AST, ALT, TC, TG, LDL, HDL, liver weight, body weight, IL-1 | Ginsenoside Rg1 | Enhances antioxidant capacity, promotes fatty acid β - oxidation | Decreases in body weight and liver wet weight, ALT, AST, and TG |
| Xu,Y et al.(2018)(26) | C57BL/6 mice (male,9/9,6-8 weeks,18-22 g) | HSFD | Gavage,20/40 mg/kg,4 weeks | Normal saline | AST, ALT, TC, TG, HDL, LDL, body weight, liver weight, IL-1 | Ginsenoside Rg1 | Upregulates the expression of PPARα | Reduces liver weight, serum ALT, AST, TG |
| Yang et al.(2023)(27) | C57BL/6 mice (male,10/10,4-6 weeks,20-25g) | HSFD and CCl4 | Gavage,60/120/180 mg/kg,8 weeks | Normal saline | AST, ALT, TC, TG, HDL, LDL, body weight, liver index, IL-1, IL-6, TNF-α, NAS score | Ginsenoside Rh4 | Modulates the FXR signaling pathway | Decreases the degrees of hepatic steatosis, lobular inflammation levels, and the content of bile acid in liver tissue |
| Yao et al.(2020)(28) | C57BL/6 mice (male,5/5,4 weeks,15-20 g) | HFD | Intraperitoneal injection,120 mg/kg/d,28 days | No mention | AST, ALT, TC, TG, LDL, HDL, body weight, liver weight, serum insulin, NAS score | Ginsenosides | Alleviates endoplasmic reticulum stress (ERS) | Inhibit appetite, reduce body weight, visceral fat, body fat content, blood glucose, and reduce glucose tolerance |
| Zhang et al.(2022)(29) | Kunming mice (male,10/10,4–6 weeks,18-20 g) | HSFD | Gavage,30/60 mg/kg,8 weeks | No mention | AST, ALT, TC, TG, HDL, LDL, liver weight, IL-6,TNF-α, NAS score | Ginsenoside CK | Activates the LKB1/AMPK pathway | Inhibit appetite, reduce body weight, visceral fat, body fat content, blood glucose, and reduce glucose tolerance |
| Zhang et al.(2024)(30) | C57BL/6 mice (male,6/6) | HFD | Gavage,20/40 mg/kg,8 weeks | Normal saline | AST, ALT, TG, HDL, LDL, body weight | Ginsenoside Re | Regulates the PI3K/AKT and TLR4/NF - κB signaling pathways | Ameliorates lipid metabolism disorders and inflammatory responses |

Supplementary Table 2 The subgroup analyses of ALT, AST, TC and TG

| Parameter | Subgroup |  | MD [95% CI] | I^2^ (%) | *P* for heterogeneity |
| --- | --- | --- | --- | --- | --- |
| ALT | Strain | mice | -35.45 [-42.22, -28.68] | 98 | <0.00001 |
|  |  | rat | -22.08 [-26.19, -17.96] | 87 | <0.00001 |
|  | Modeling methods | HFD | -37.00 [-45.26, -28.75] | 98 | <0.00001 |
|  |  | HSFD | -23.23 [-27.56, -18.90] | 90 | <0.00001 |
|  |  | HSFD and CCl4 | -38.77 [-61.98, -15.55] | 98 | <0.00001 |
|  | Administration method | Intraperitoneal injection | -49.04 [-74.89, -23.18] | 99 | <0.00001 |
|  |  | Gavage | -25.58 [-30.38, -20.78] | 96 | <0.00001 |
|  | Treatment dose (mg/kg) | >40 | -28.07 [-34.86, -21.28] | 97 | <0.00001 |
|  |  | ≤40 | -32.07 [-39.54, -24.59] | 98 | <0.00001 |
|  | Types of ginsenosides | PPD | -48.24 [-64.13, -32.36] | 98 | <0.00001 |
|  |  | PPT | -27.15 [-32.90, -21.40] | 98 | <0.00001 |
|  |  | Other | -27.73 [-47.50, -7.96] | 95 | <0.00001 |
| AST | Strain | mice | -38.04 [-53.66, -22.42] | 100 | <0.00001 |
|  |  | rat | -62.97 [-85.35, -40.59] | 99 | <0.00001 |
|  | Modeling methods | HFD | -40.37 [-72.41, -8.33] | 100 | <0.00001 |
|  |  | HSFD | -52.89 [-68.61, -37.16] | 99 | <0.00001 |
|  |  | HSFD and CCl4 | -49.30 [-75.96, -22.64] | 94 | <0.00001 |
|  | Administration method | Intraperitoneal injection | -34.17 [-62.05, -6.29] | 100 | <0.00001 |
|  |  | Gavage | -51.48 [-63.04, -39.92] | 98 | <0.00001 |
|  | Treatment dose (mg/kg) | >40 | -51.27 [-69.19, -33.34] | 99 | <0.00001 |
|  |  | ≤40 | -42.75 [-52.75, -32.75] | 99 | <0.00001 |
|  | Types of ginsenosides | PPD | -28.06 [-37.57, -18.55] | 96 | <0.00001 |
|  |  | PPT | -54.35 [-66.85, -41.85] | 99 | <0.00001 |
|  |  | Other | -45.89 [-100.89, B7:F89.11] | 99 | <0.00001 |
| TC | Strain | mice | -1.18 [-1.53, -0.82] | 97 | <0.00001 |
|  |  | rat | -0.63 [-0.74, -0.52] | 82 | <0.00001 |
|  | Modeling methods | HFD | -1.11 [-1.52, -0.70] | 97 | <0.00001 |
|  |  | HSFD | -0.72 [-0.86, -0.57] | 91 | <0.00001 |
|  |  | HSFD and CCl4 | -1.53 [-2.67, -0.39] | 67 | 0.08 |
|  | Administration method | Intraperitoneal injection | -1.39 [-1.98, -0.81] | 98 | <0.00001 |
|  |  | Gavage | -0.77 [-0.93, -0.60] | 94 | <0.00001 |
|  | Treatment dose (mg/kg) | >40 | -0.84 [-1.08, -0.59] | 96 | <0.00001 |
|  |  | ≤40 | -1.00 [-1.29, -0.71] | 96 | <0.00001 |
|  | Types of ginsenosides | PPD | -0.75 [-1.10, -0.41] | 97 | <0.00001 |
|  |  | PPT | -1.06 [-1.29, -0.82] | 96 | <0.00001 |
|  |  | Other | -1.13 [-1.98, -0.27] | 80 | 0.007 |
| TG | Strain | mice | -0.31 [-0.39, -0.23] | 97 | <0.00001 |
|  |  | rat | -0.59 [-0.85, -0.32] | 99 | <0.00001 |
|  | Modeling methods | HFD | -0.28 [-0.36, -0.20] | 97 | <0.00001 |
|  |  | HSFD | -0.57 [-0.82, -0.33] | 99 | <0.00001 |
|  |  | HSFD and CCl4 | -0.40 [-0.62, -0.19] | 86 | 0.0008 |
|  | Administration method | Intraperitoneal injection | -0.42 [-0.58, -0.25] | 98 | <0.00001 |
|  |  | Gavage | -0.46 [-0.61, -0.30] | 99 | <0.00001 |
|  | Treatment dose (mg/kg) | >40 | -0.59 [-0.80, -0.38] | 99 | <0.00001 |
|  |  | ≤40 | -0.29 [-0.37, -0.22] | 94 | <0.00001 |
|  | Types of ginsenosides | PPD | -0.24 [-0.32, -0.16] | 92 | <0.00001 |
|  |  | PPT | -0.54 [-0.76, -0.31] | 99 | <0.00001 |
|  |  | Other | -0.55 [-0.84, -0.26] | 97 | <0.00001 |

**References**

1. Cui TQ, Xiao XX, Pan ZS, Tang KJ, Zhong YD, Chen YJ, et al. Harnessing the Therapeutic Potential of Ginsenoside Rd for Activating Sirt6 in Treating a Mouse Model of Nonalcoholic Fatty Liver Disease. *Acs Omega* (2023) 8(32):29735-45. doi: 10.1021/acsomega.3c04122.

2. Cui T. Research on the activation of sirt6 by ginsenoside rd to improve non-alcoholic fatty liver disease induced by high-fat diet. *Guangzhou University of Chinese Medicine* (2024). (In Chinese).

3. Gao X, Zhang L, Yang Y, Luo J, Jiang K, Li S, et al. Effect of ginsenoside Rg1 on non-alcoholic fatty liver disease and intestinal microbiota in rats. *Journal of Chongqing Medical University* (2023) 48(04):381-9. doi: 10.13406/j.cnki.cyxb.003212. (In Chinese).

4. Gu DS, Yi HA, Jiang KR, Fakhar SH, Shi J, He YS, et al. Transcriptome Analysis Reveals the Efficacy of Ginsenoside-Rg1 in the Treatment of Nonalcoholic Fatty Liver Disease. *Life Sciences* (2021) 267. doi: 10.1016/j.lfs.2020.118986.

5. Guo MD, Zhu CH, Fu RZ, Ma XX, Duan ZG, Fan DD. Ginsenoside Rk3 Regulates Nonalcoholic Steatohepatitis by Modulation of Intestinal Flora and the Pi3k/Akt Signaling Pathway in C57bl/6 Mice. *Journal of Agricultural and Food Chemistry* (2023) 71(24):9370-80. doi: 10.1021/acs.jafc.3c00789.

6. Han X, Zhang F, Liu C, Zhang H, Gu L, Liu Y, et al. Therapeutical effect of ginsenoside Rb1 on non-alcoholic fatty liver disease of mice. *Shandong Medical Journal* (2019) 59(08):37-40. (In Chinese).

7. He W, Yang R, Luo T. Effect of Ginsenoside Rg1 on Liver Fibrosis in Mice with Non-alcoholic Fatty Liver Disease. *Journal of Chinese Medicinal Materials* (2021) 44(05):1208-12. doi: 10.13863/j.issn1001-4454.2021.05.032. (In Chinese).

8. Hong Y, Gu X, Xu J, Lin Y, Si Q. The effect and mechanism of ginsenoside rb2 on lipid metabolism in the liver of mice with hyperlipidemic fatty liver. *Journal of Wenzhou Medical University* (2018) 48(05):338-41+49. (In Chinese).

9. Hou YH, Gu DS, Peng JZ, Jiang KR, Li ZG, Shi J, et al. Ginsenoside Rg1 Regulates Liver Lipid Factor Metabolism in Nafld Model Rats. *Acs Omega* (2020) 5(19):10878-90. doi: 10.1021/acsomega.0c00529.

10. Huang Q, Wang T, Yang L, Wang HY. Ginsenoside Rb2 Alleviates Hepatic Lipid Accumulation by Restoring Autophagy Via Induction of Sirt1 and Activation of Ampk. *International Journal of Molecular Sciences* (2017) 18(5). doi: 10.3390/ijms18051063.

11. Jiang K. Study on the Molecular Mechanism of Ginsenoside rg1 Regulating Early Liver Fibrosis in Non-Alcoholic Fatty Liver Disease. *Kunming Medical University* (2021). (In Chinese).

12. Li CY, Fan DJ. Ginsenoside R3 Alleviates Non-Alcoholic Fatty Liver Disease by Regulating the Pparγ/Mir-103-3p Pathway. *Tropical Journal of Pharmaceutical Research* (2024) 23(8):1239-47. doi: 10.4314/tjpr.v23i8.2.

13. Li N, Zhu CH, Fu RZ, Ma XX, Duan ZG, Fan DD. Ginsenoside Rg5 Inhibits Lipid Accumulation and Hepatocyte Apoptosis Via the Notch1 Signaling Pathway in Nash Mice. *Phytomedicine* (2024) 124. doi: 10.1016/j.phymed.2023.155287.

14. Liang W, Zhou K, Jian P, Chang Z, Zhang Q, Liu Y, et al. Ginsenosides Improve Nonalcoholic Fatty Liver Disease Via Integrated Regulation of Gut Microbiota, Inflammation and Energy Homeostasis. *Front Pharmacol* (2021) 12:622841. Epub 2021/03/09. doi: 10.3389/fphar.2021.622841.

15. Liang Y, Fu J, Shi Y, Gao X, Lu F, Liu S. Integrating network pharmacology and experimental verification to explore mechanism of ginsenoside Rb in treating metabolic associated fatty liver disease. *Drug Evaluation Research* (2023) 46(12):2580-91. (In Chinese).

16. Liao W, Xu D. Study on the Improvement Effect of Ginsenoside rg1 on Fatty Acid β -Oxidation in Rats with Non-Alcoholic Fatty Liver Disease. *Chongqing Medical Journal* (2016) 45(09):1179-82. (In Chinese).

17. Peng X, Huang D, Yan M, Peng S. Ginsenoside Rg1 improves liver function by regulating fat metabolism in rats with non-alcoholic fatty liver disease. *Chinese Journal of Pathophysiology* (2015) 31(05):864-70. (In Chinese).

18. Qi R, Jiang R, Xiao H, Wang Z, He S, Wang L, et al. Ginsenoside Rg1 Protects against D-Galactose Induced Fatty Liver Disease in a Mouse Model Via Foxo1 Transcriptional Factor. *Life Sci* (2020) 254:117776. Epub 2020/05/22. doi: 10.1016/j.lfs.2020.117776.

19. Ruan J, Xiao T, Chen J, Meng X, Song H, Wang B. Research of Mechanisms of Ginsenosides Adjusting Glycolipid Metabolism in Nonalcoholic Fatty Liver Disease Rats. *Chinese Archives of Traditional Chinese Medicine* (2020) 38(08):101-6+269. doi: 10.13193/j.issn.1673-7717.2020.08.025. (In Chinese).

20. Shi Y, Chen J, Qu D, Sun Q, Yu Y, Zhang H, et al. Ginsenoside Rg(5) Activates the Lkb1/Ampk/Mtor Signaling Pathway and Modifies the Gut Microbiota to Alleviate Nonalcoholic Fatty Liver Disease Induced by a High-Fat Diet. *Nutrients* (2024) 16(6). Epub 2024/03/28. doi: 10.3390/nu16060842.

21. Song B, Sun Y, Chu YF, Wang J, Zheng HW, Liu LL, et al. Ginsenoside Rb1 Alleviated High-Fat-Diet-Induced Hepatocytic Apoptosis Via Peroxisome Proliferator-Activated Receptor Γ. *Biomed Research International* (2020) 2020. doi: 10.1155/2020/2315230.

22. Wang K, Hu H, Xu Y, Ding R, Wang Y, Duan Z. Effect of ginsenoside Rb2 on nonalcoholic fatty liver disease in mice and its mechanism. *Journal of Tropical Medicine* (2021) 21(02):125-9+43+0. (In Chinese).

23. Xiao Y, Hou Y, Yin X, Kang F, Li S, Yang S, et al. Ginsenoside rg1 intervenes in the apoptosis of hepatocytes in rat models of non-alcoholic fatty liver disease. *Chinese Journal of Tissue Engineering Research* (2019) 23(03):384-90. (In Chinese).

24. Xie J, Li D, Liu C, Xiao X. The intervention effect of ginsenoside Rg1 on rats with non-alcoholic fatty liver disease. *Chinese Traditional Patent Medicine* (2023) 45(11):3799-802. (In Chinese).

25. Xu YS, Yang C, Zhang SJ, Li JJ, Xiao Q, Huang WX. Ginsenoside Rg1 Protects against Non-Alcoholic Fatty Liver Disease by Ameliorating Lipid Peroxidation, Endoplasmic Reticulum Stress, and Inflammasome Activation. *Biological & Pharmaceutical Bulletin* (2018) 41(11):1638-44. doi: 10.1248/bpb.b18-00132.

26. Xu Y. Study on the improvement effect and mechanism of Ginsenoside Rg1 on Non-alcoholic Fatty Liver Disease in Mice. *Chongqing Medical University* (2018) (In Chinese).

27. Yang SM, Duan ZG, Zhang S, Fan CY, Zhu CH, Fu RZ, et al. Ginsenoside Rh4 Improves Hepatic Lipid Metabolism and Inflammation in a Model of Nafld by Targeting the Gut Liver Axis and Modulating the Fxr Signaling Pathway. *Foods* (2023) 12(13). doi: 10.3390/foods12132492.

28. Yao Y. Ginsenosides Reduce Body Weight and Ameliorate Hepatic Steatosis in High Fat Diet‑Induced Obese Mice Via Endoplasmic Reticulum Stress and P‑Stat3/Stat3 Signaling. *Mol Med Rep* (2020) 21(3):1059-70. Epub 2020/02/06. doi: 10.3892/mmr.2020.10935.

29. Zhang JJ, Ma XX, Fan DD. Ginsenoside Ck Ameliorates Hepatic Lipid Accumulation <I>Via</I> Activating the Lkb1/Ampk Pathway <I>in Vitro</I> and <I>in Vivo</I>. *Food & Function* (2022) 13(3):1153-67. doi: 10.1039/d1fo03026d.

30. Zhang J, Duan M, Wu S, Jiang S, Hu S, Chen W, et al. Comprehensive Pharmacological and Experimental Study of Ginsenoside Re as a Potential Therapeutic Agent for Non-Alcoholic Fatty Liver Disease. *Biomedicine and Pharmacotherapy* (2024) 177. doi: 10.1016/j.biopha.2024.116955.
